# Probing the molecular basis for signal transduction through the Zinc-Activated Channel (ZAC)

**DOI:** 10.1016/j.bcp.2021.114781

**Published:** 2021-09-21

**Authors:** Nawid Madjroh, Eleni Mellou, Laura Æbelø, Paul A. Davies, Pella C. Söderhielm, Anders A. Jensen

**Affiliations:** a Department of Drug Design and Pharmacology, Faculty of Health and Medical Sciences, University of Copenhagen, Copenhagen Ø 2100, Denmark; b Department of Neuroscience, Tufts University School of Medicine, Boston, MA, United States

**Keywords:** Cys-loop receptor (CLR), Pentameric ligand-gated ion channel (pLGIC), Zinc-Activated Channel (ZAC), Agonist binding, Chimeric subunits, Leu9′ residue

## Abstract

The molecular basis for the signal transduction through the classical Cys-loop receptors (CLRs) has been delineated in great detail. The Zinc-Activated Channel (ZAC) constitutes a so far poorly elucidated fifth branch of the CLR superfamily, and in this study we explore the molecular mechanisms underlying ZAC signaling in *Xenopus* oocytes by two-electrode voltage clamp electrophysiology. In studies of chimeric receptors fusing either the extracellular domain (ECD) or the transmembrane/intracellular domain (TMD-ICD) of ZAC with the complementary domains of *5*-HT*3*A serotonin or α_1_ glycine receptors, serotonin and Zn^2+^/H^+^ evoked robust concentration-dependent currents in *5*-HT*3*A/ZAC- and ZAC/α_1_-Gly-expressing oocytes, respectively, suggesting that Zn^2+^ and protons activate ZAC predominantly through its ECD. The molecular basis for Zn^2+^-mediated ZAC signaling was probed further by introduction of mutations of His, Cys, Glu and Asp residues in this domain, but as none of the mutants tested displayed substantially impaired Zn^2+^ functionality compared to wild-type ZAC, the location of the putative Zn^2+^ binding site(s) in the ECD was not identified. Finally, the functional importance of Leu^246^ (Leu9′) in the transmembrane M2 α-helix of ZAC was investigated by Ala, Val, Ile and Thr substitutions. In concordance with findings for this highly conserved residue in classical CLRs, the ZAC^L9′x^ mutants exhibited left-shifted agonist concentration-response relationships, markedly higher degrees of spontaneous activity and slower desensitization kinetics compared to wild-type ZAC. In conclusion, while ZAC is an atypical CLR in terms of its (identified) agonists and channel characteristics, its signal transduction seems to undergo similar conformational transitions as those in the classical CLR.

## 1. Introduction

The Cys-loop receptor (CLR) superfamily contains pentameric ligand-gated ion channels that mediate the fast signalling of the important neurotransmitters acetylcholine (ACh), serotonin (5-hydroxytryptamine, 5-HT), γ-aminobutyric acid (GABA) and glycine (Gly). The nicotinic ACh, 5-HT_3_, GABA_A_ and Gly receptors (nAChRs, 5-HT_3_RS, GABA_A_Rs and GlyRs, respectively) govern a plethora of important physiological functions and are implicated in numerous pathophysiological disorders, and these classical CLRs thus constitute therapeutic targets for numerous indications [1-9].

The CLR is a homomeric or heteromeric complex assembled from five subunits and comprises three distinct structural domains. The extracellular domain (ECD) is composed by the β-sheets β1-β10 and interconnecting loops in the *N*-termini of the five subunits, the transmembrane domain (TMD) is constituted by the four transmembrane α-helices (M1-M4) from each of the five subunits, and the intracellular domain (ICD) is mainly composed by the second intracellular loops from the five subunits [10-18]. Signal transduction through the classical CLR is initiated by agonist binding to the orthosteric site formed by three loops from each of two neighboring subunits (loops A-C and D-F) in the ECD. The conformational changes in the ECD triggered by this agonist binding facilitates cross-talk between the ECD and TMD via interactions between three ECD loops [β1-β2, β6-β7 (Cys-loop), β8-β9] and the extracellular M2-M3 linker in the TMD. This in turn causes an outward rotation of the ion pore-lining M2 α-helix in the TMD leading to opening of the ion channel and the flux of ions through it. The CLR exists in this active agonist-bound/open state until it deactivates (agonist unbinding and a return to its resting unbound/closed state) or desensitizes (collapse of the agonist-bound/open channel into an agonist-bound/closed state) [10-16,19]. The diverse kinetic properties exhibited by different CLRs are rooted in the different energy barriers associated with the transitions of the receptors through their resting, active and desensitized states [12,16,20,21].

With the discovery of the Zinc-Activated Channel (ZAC) in 2003, the CLR superfamily was extended with a distinct fifth mammalian receptor subfamily [22]. Although the ZAC protein shares very low amino acid sequence homology with the classical CLRs, it comprises most of the structural hallmarks for a CLR subunit. Moreover, when expressed in mammalian cells or *Xenopus* oocytes ZAC assembles into functional homomeric cation-selective channels gated by zinc (Zn^2+^), copper (Cu^2+^) and protons (H^+^) [22-24]. ZAC has been found to be expressed at the transcript level in several human organs [22,25,26], and the agonists identified for ZAC so far could be indicative of a role for the channel as an *in vivo* sensor of changes in transient divalent metal ion concentrations and/or in pH. However, presently very little is known about the putative physiological functions of the receptor.

ZAC is an atypical CLR, both when it comes to its identified agonists and its channel characteristics. Although Zn^2+^, Cu^2+^ and H^+^ are known to modulate the signalling through several of the classical CLRs via various allosteric sites [27-40] and proton-gated CLRs have been identified in bacteria, *C. elegans* and *D. melanogaster* [41-43], the direct gating mediated by these ions is unique for ZAC amongst the mammalian CLRs. In addition to its distinct agonists, the gating characteristics exhibited by homomeric ZAC in heterologous expression systems also differ substantially from those displayed by classical CLRs [22-24]. Thus, ZAC has exhibited substantial levels of spontaneous activity, slow activation and desensitization kinetics, and low degrees of desensitization at subsaturating agonist concentrations in both patch-clamp recordings from HEK293 and COS-7 cells [22,23] and in two-electrode voltage-clamp (TEVC) recordings from *Xenopus* oocytes [24].

In the present work, we aimed to elucidate the molecular mechanisms underlying ZAC signaling and shed light on similarities and differences in its signal transduction compared to that of the classical CLR. To address this, we compared the functional properties exhibited by wild-type (WT) ZAC with those displayed by chimeric receptors fusing the ECD or TMD-ICD of ZAC with the complementary domains of two classical CLRs and by various ZAC mutants expressed in *Xenopus* oocytes by TEVC electrophysiology.

## 2. Materials and methods

### 2.1. Materials

ZnCl_2_, 5-HT, glycine, and all chemicals for the buffers were purchased from Sigma-Aldrich (St. Louis, MO), picrotoxin (PTX) and tubocurarine (TC) were purchased from Tocris Cookson (Bristol, UK), and antibiotics were obtained from Invitrogen (Paisley, UK). PfuUltra II High-fidelity DNA Polymerase was purchased from Stratagene (Santa Clara, CA), restriction enzymes were obtained from New England Biolabs (Ipswich, MA), and oligonucleotides were obtained from TAG Copenhagen (Frederiksberg, Denmark). The original cDNAs for the human ZAC, the mouse 5-HT3A (m5-HT3A) and human α_1_ (hα_1_) GlyR subunits were kind gifts from Drs. E. Kirkness, D. Julius and P.R. Schofield, respectively. Defolliculated stage V-VI oocytes harvested from female *Xenopus laevis* frogs were obtained from Lohmann Research Equipment (Castrop-Rauxel, Germany) and from an in-house facility. The care and use of *Xenopus laevis* from the in-house facility was in strict adherence to a protocol (license 2014-15-0201-00031) approved by the Danish Veterinary and Food Administration, in accordance with the Guide for the Care and Use of Laboratory Animals adopted by the U.S. National Institutes of Health.

### 2.2. Molecular biology

The construction of the ZAC-pUNIV plasmid (encoding for the human WT ZAC that contains a Thr in position 128) has been described previously [24]. The m5-HT3A and hα_1_ GlyR cDNAs were subcloned from their original vectors into pUNIV (Addgene, Watertown, MA) using *Nhe*I and *EcoR*I as restriction enzymes. The cDNAs for the chimeric ZAC/m5-HT_3_A, m5-HT_3_A/ZAC, ZAC/hα_1_-Gly, hα_1_-Gly/ZAC, ZAC/m5-HT_3_A-II and hα_1_-Gly/ZAC-II subunits were constructed using the splicing by overlap extension PCR technique [44] and subcloned into the pUNIV vector using *Nhe*I and *EcoR*I as restriction enzymes. The C-terminus modifications in these four chimeras were introduced in their cDNAs in the same way. Point mutations were introduced in cDNAs by use of the QuikChange mutagenesis kit (Stratagene, San Diego, CA). The validity and absence of unwanted mutations in the constructed cDNAs was verified by DNA sequencing (Macrogen Europe, Amsterdam, The Netherlands).

### 2.3. Xenopus oocytes and two-electrode voltage clamp (TEVC) recordings

All cDNAs used for cRNA synthesis were inserted in the pUNIV vector. The cDNAs were linearized and subsequently transcribed and capped using the mMessage mMachine T7 RNA transcription kit (Ambion, Waltham, MA). Volumes of 4.6–36.8 nL cRNA solution and the following cRNA amounts were injected into the oocytes: 1.15 ng (WT ZAC, WT m5-HT_3_AR, WT hα_1_ GlyR), 3.60 ng (m5-HT_3_A/ZAC, ZAC/hα_1_-Gly/ZAC), 4.32 ng (ZAC/m5-HT_3_A, ZAC/m5-HT_3_A-II, hα_1_-Gly/ZAC, hα_1_-Gly/ZAC-II), and 2.88–3.60 ng (the four ZAC^L9′X^ mutants) for the experiments described in sections 3.1 and 3.3, and 1.84 ng (WT ZAC and ZAC mutants) for the experiments described in Section 3.2. Oocytes were incubated in a sterile modified Barth’s solution [88 mM NaCl, 1 mM KCl, 15 mM HEPES (pH 7.5), 2.4 mM NaHCO_3_, 0.41 mM CaCl_2_, 0.82 mM MgSO_4_, 0.3 mM Ca(NO_3_)_2_, 100 U/ml penicillin and 100 μg/ml streptomycin] at 16–18 °C. Based on experience gained with WT ZAC in a recent study [24], all TEVC recordings were performed two days after cRNA injection.

On the day of the TEVC recording, all compound dilutions were prepared in a saline solution [115 mM NaCl, 2.5 mM KCl, 10 mM MOPS (pH 7.5), 1.8 mM CaCl_2_, 0.1 mM MgCl_2_], and pH was adjusted to 7.5 (if needed). Oocytes were placed in a recording chamber continuously perfused with this saline solution, and the compounds were applied in the perfusate. Both voltage and current electrodes were agar-plugged with 3 M KCl with a resistance of 0.2–2.0 MΩ. Oocytes were voltage-clamped at −50 mV (except in the studies of the chimeras and their parent receptors where they were clamped at −60 mV, section 3.1), by a Gene Clamp 500B amplifier, and current signals were digitized by a Digidata 1322A (both from Axon Instruments, Union City, CA). Currents were recorded using pCLAMP 10 (Molecular Devices, Sunnyvale, CA). The recordings were performed at room temperature.

In all recordings, compounds or compound combinations were applied in the bath until the peak current decayed to a steady state (up to 30 s). As also observed in our recent study of ZAC in oocytes [24], the currents evoked by sub-saturating concentrations of Zn^2+^ and H^+^ at ZAC did not reach well-defined peaks during the 30-s application, but the pharmacological properties displayed by the agonists at the receptor were nevertheless reflected well by the data extracted from these recordings. At the beginning or end (whenever appropriate) of all recordings determining concentration–response relationships for agonists or concentration-inhibition relationships for antagonists, two consecutive applications of an agonist concentration giving rise to a maximal current (I_max_) at the specific receptor were applied on the oocyte, and it was verified that these consecutive applications elicited responses of comparable current amplitudes (±20%). The antagonist properties of compounds were determined by pre-application of the compound to the perfusate for 30 s followed by co-application of the compound and the agonist. In all recordings, washes of 1–5 min were executed between the ligand applications, the length of the washes depending on the agonist concentrations used and on the return to baseline current amplitude.

### 2.4. Data and statistical analysis

Analysis of the data from the TEVC recordings were performed using Clampfit software version 10.5 (Molecular Devices, Crawley, UK) and GraphPad Prism version 7.0c (GraphPad Software, La Jolla, CA). Unless otherwise stated, the inward currents induced by agonists in oocytes were normalized to the maximal response elicited by a specific agonist (agonist I_max_) on each oocyte. Concentration-response and concentration-inhibition curves were fitted in GraphPad Prism by nonlinear regression using the equation for sigmoidal dose-response with variable slope. Each data point represents the mean ± S.E.M. value of recordings performed on at least five oocytes in total from at least two different batches. For the data where statistical analysis was performed, a one-way ANOVA was used. The null hypothesis was rejected at *P* < 0.05, and the differences between the means were analyzed by Tukey’s multiple comparisons test.

### 2.5. Homology model of ZAC

The construction of the homology model of the pentameric ZAC complex used in this study has been described previously [24]. The model was created based on an alignment of the amino acid sequences of the ZAC and m5-HT3A subunits in the Chimera software [45]. The homology model was not refined further, and in the present work the model is thus exclusively used to get an idea of the approximate locations of specific residues in the ZAC ECD and for illustration purposes.

## 3. Results

### 3.1. Functional characterization of chimeric receptors fusing ECD and TMD-ICD from ZAC and classical CLRs

As outlined in Section 1, the agonist-induced conformational changes in the ECD of the CLR are translated into gating of the ion channel in the TMD of the pentamer via interactions between residues in the two domains. Despite this specificity of the molecular interactions underlying this inter-domain cross-talk in each CLR, several chimeric CLR subunits fusing the ECD of one CLR with the TMD and ICD of another have been shown to be able to form functional receptors. Such functional chimeras have not only emerged from fusions of ECDs and TMD-ICDs from orthologous subunits from different species or from closely related subunits within the same CLR subfamily [46-51], but also from fusions of domains from subunits from different CLR subfamilies (e.g., α_7_nACh/5-HT_3_A and ρ_1_-GABA_A_/α_1_-Gly chimeras) [52-55] and of domains from very distantly related eukaryotic CLRs and prokaryotic CLRs (such as *Gloeobacter violaceus* and *Erwinia chrysanthemi* ion channels, GLIC and ELIC) [56-59]. This inspired us to use this chimeric approach to elucidate the signal transduction in ZAC.

#### 3.1.1. Construction and characterization of the initial series of ECD/TMD-ICD chimeras

**Fig. 1.**
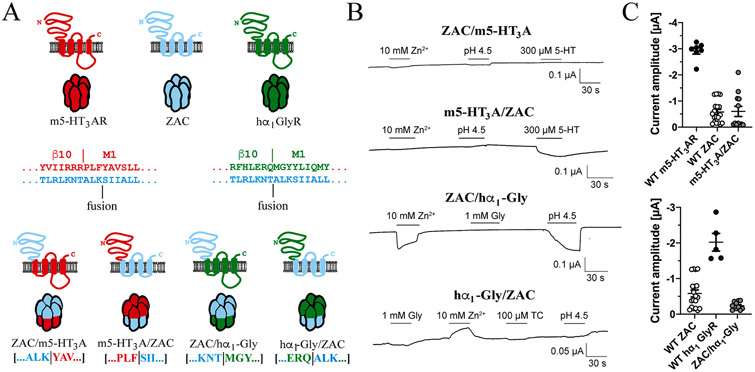
Chimeric receptors fusing the ECDs and TMD-ICDs of ZAC, m5-HT_3_AR and hα_1_ GlyR. **A.** Topologies of WT m5-HT3A, WT ZAC and WT hα_1_ GlyR subunits and chimeric ZAC/m5-HT_3_A, m5-HT_3_A/ZAC, ZAC/hα_1_-Gly and hα_1_-Gly/ZAC subunits and illustration of the pentameric complexes assembled from them. The amino acid sequences of the ECD-into-TMD sequences in the three WT subunit proteins are given. The borders between β10 (ECD) and M1 (TMD) reported for m5-HT_3_AR [11] and hα_1_ GlyR [15] and the fusion points in the four chimeras (“fusion”) are indicated. **B.** Functionalities of the constructed chimeric receptors. Representative traces from the testing of the putative agonists at ZAC/m5-HT_3_A-, m5-HT_3_A/ZAC-, ZAC/hα_1_-Gly- and hα_1_-Gly/ZAC-expressing oocytes by TEVC electrophysiology. **C.** Averaged agonist-evoked current amplitudes in oocytes expressing the functional m5-HT_3_A/ZAC (*top*) and ZAC/hα_1_-Gly (*bottom*) chimeras and their respective parent receptors. Saturating agonist concentrations for the different receptors were used: 300 μM 5-HT (WT m5-HT_3_AR), 3 μM 5-HT (m5-HT_3_A/ZAC), 10 mM Zn^2+^ (WT ZAC), 30 μM Zn^2+^ (ZAC/hα_1_-Gly), and 1 mM Gly (WT hα_1_ GlyR). The averaged data are given as means ± S.E.M. and are based on data from recordings performed on the receptors expressed in at least two diffent oocyte batches (n = 5–19).

Initially, we constructed four chimeric subunits fusing the ZAC ECD or TMD-ICD with the complementary domains from either the m5-HT3A or the hα_1_ GlyR subunit: ZAC/m5-HT_3_A, m5-HT_3_A/ZAC, ZAC/hα_1_-Gly and hα_1_-Gly/ZAC (Fig. 1A). The fusion points between ECD and TMD-ICD in the ZAC/m5-HT_3_A and m5-HT_3_A/ZAC chimeras corresponded to that in a previously published functional m5-HT_3_A/ELIC chimera [58], whereas the fusion points in the ZAC/hα_1_-Gly and hα_1_-Gly/ZAC chimeras corresponded to that in a previously published functional GLIC/α_1_-Gly chimera [57] (Fig. 1A). The four chimeric subunits were expressed in *Xenopus* oocytes, and the effects of applications of 5-HT (300 μM) at ZAC/m5-HT_3_A and m5-HT_3_A/ZAC, of Gly (1 mM) at ZAC/hα_1_-Gly and hα_1_-Gly/ZAC, and of Zn^2+^ (10 mM) and H^+^ (pH 4.5) at all four chimeras were investigated by TEVC recordings (Fig. 1B). The agonist concentrations tested at the chimeras were chosen based them being high (H^+^) or saturating (Zn^2+^, 5-HT, Gly) concentrations at their respective “parent” receptors (WT ZAC, WT m5-HT_3_AR, WT hα_1_ GlyR). The inclusion of 5-HT (300 μM) and Gly (1 mM) in the testing of ZAC/m5-HT_3_A and ZAC/hα_1_-Gly, respectively, was mainly done for reasons of consistency, since these orthosteric agonists of m5-HT_3_AR and hα_1_ GlyR were not expected to gate these chimeras.

The ZAC/m5-HT_3_A chimera was found to be non-functional, as neither Zn^2+^ nor H^+^ evoked significant currents in oocytes expressing this subunit (I_10 mM Zn2+_ ± S.E.M.: −0.0038 ± 0.0009 μA; I_pH 4.5_ ± S.E. M.: −0.0033 ± 0.0003 μA; both n = 5, Fig. 1B). We did not investigate whether the chimera was expressed at the oocyte cell surface. Interestingly, 5-HT evoked inward currents of substantial amplitudes in m5-HT_3_A/ZAC-expressing oocytes (Fig. 1B and 1C). In contrast, neither Zn^2+^ nor H^+^ elicited significant currents in the m5-HT_3_A/ZAC-oocytes (I_10 mM Zn2+_ ± S.E.M.: 0.0037 ± 0.0013 μA, I_pH 4.5_ ± S.E.M.: −0.0027 ± 0.0007 μA; both n = 4, Fig. 1B). Both Zn^2+^ and H^+^ elicited robust currents in ZAC/hα_1_-Gly-expressing oocytes (Fig. 1B and 1C). Finally, whereas applications of H^+^ or Gly did not mediate significant currents in hα_1_-Gly/ZAC-expressing oocytes (I_pH 4.5_ ± S.E.M.: −0.0045 ± 0.0019 μA; I_1 mM Gly_ ± S.E.M.: −0.0062 ± 0.0022 μA; both n = 5), Zn^2+^ was observed to produce small but significant outward currents in these oocytes (I_10 mM Zn2+_ ± S.E.M.: 0.0298 ± 0.0058 μA; n = 5, Fig. 1B). Thus, out of the four chimeras, only m5-HT_3_A/ZAC and ZAC/hα_1_-Gly were functional, in the sense that 5-HT or Zn^2+^/H^+^ evoked significant inward currents through them, and thus the functional properties exhibited by these two receptors were characterised in further detail (Sections 3.1.2 and 3.1.3).

#### 3.1.2. Functional characterization of the m5-HT_3_A/ZAC chimera

**Fig. 2.**
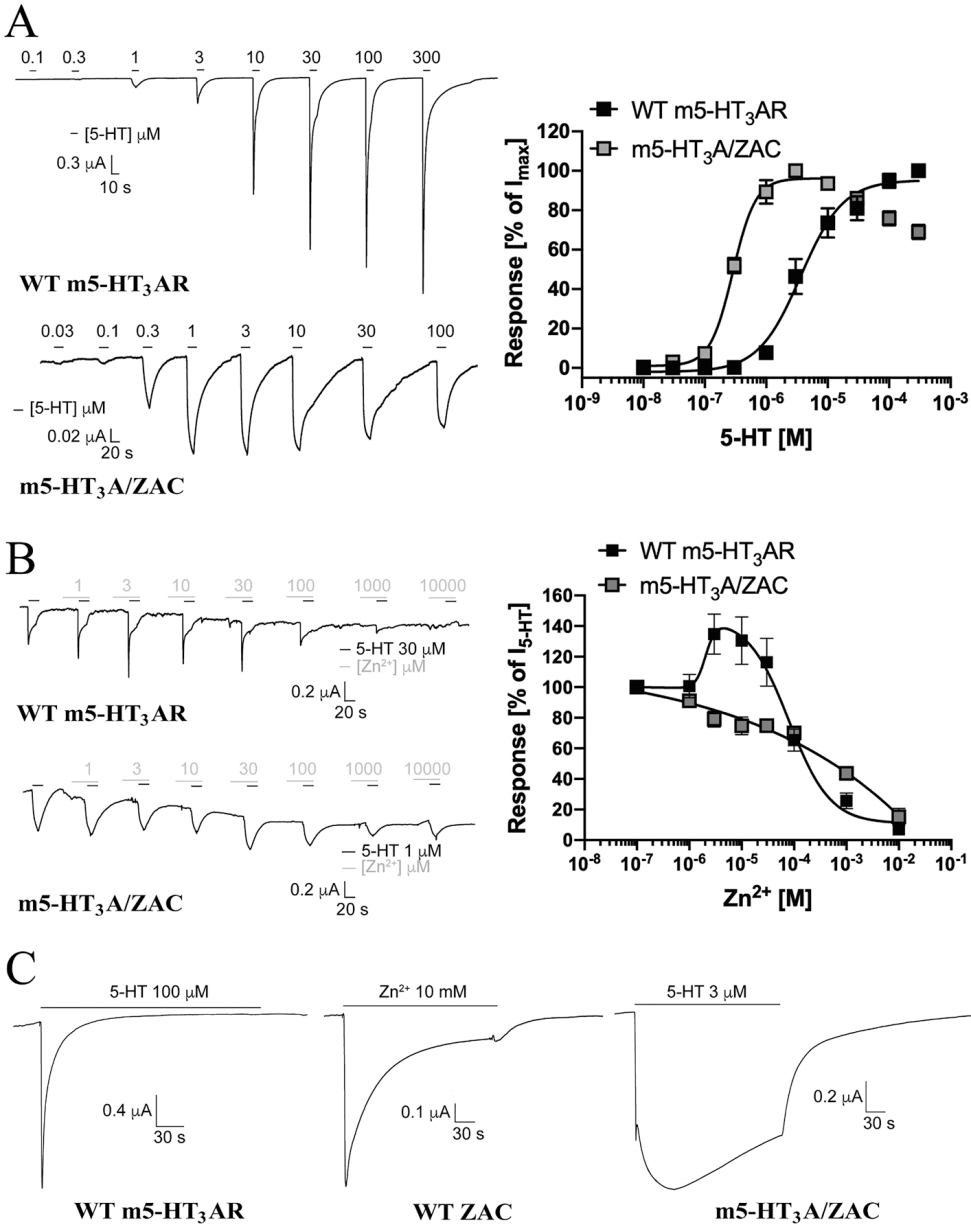
Functional properties exhibited by the chimeric m5-HT_3_A/ZAC receptor. **A.** Agonist properties displayed by 5-HT at m5-HT_3_A/ZAC. Representative traces for 5-HT-evoked currents in WT m5-HT_3_AR- and m5-HT_3_A/ZAC-expressing oocytes (*left*) and averaged concentration-response relationships displayed by 5-HT at WT m5-HT_3_AR and m5-HT_3_A/ZAC (means ± S.E.M., n = 7–9) (*right*). **B.** Modulatory properties displayed by Zn^2+^ at m5-HT_3_A/ZAC. Representative traces for the Zn^2+^-mediated modulation of 5-HT (EC_80_)-evoked currents in WT m5-HT_3_AR- and m5-HT_3_A/ZAC-expressing oocytes (*left*) and averaged concentration-effect relationships displayed by Zn^2+^ at the 5-HT (EC_80_)-mediated currents through WT m5-HT_3_AR and m5-HT_3_A/ZAC (means ± S.E.M., n = 7–8) (*right*). 5-HT (30 μM) and 5-HT (1 μM) were used for WT m5-HT_3_AR and m5-HT_3_A/ZAC, respectively. **C.** Representative traces of the currents evoked by 4 min-applications of saturating agonist concentrations at WT m5-HT_3_AR-, WT ZAC- and m5-HT_3_A/ZAC-expressing oocytes. 5-HT (100 μM), Zn^2+^ (10 mM) and 5-HT (3 μM) were used at WT m5-HT_3_AR, WT ZAC and m5-HT_3_A/ZAC, respectively.

Although oocytes were injected with a 3-fold higher quantity of cRNA for m5-HT_3_A/ZAC than for WT m5-HT_3_AR and WT ZAC, the averaged current amplitudes evoked by 5-HT in the m5-HT_3_A/ZAC-oocytes (I_max_: 0.60 ± 0.19 μA, n = 12) were substantially smaller than those in WT m5-HT_3_AR-oocytes (I_max_: 2.92 ± 0.13 μA, n = 7) and comparable to those evoked by Zn^2+^ in WT ZAC-oocytes (I_max_: 0.58 ± 0.10 μA, n = 19) (Fig. 1C). Interestingly, the functional expression levels of the chimeric receptor in the oocytes varied considerably, with the m5-HT_3_A/ZAC-oocytes grouping into two halfs characterized by 5-HT-evoked current amplitudes of 0.1–0.2 μA and 0.8–2.1 μA, respectively (Fig. 1C). Importantly, however, the functional properties and signalling characteristics exhibited by m5-HT_3_A/ZAC in these low-expressing and high-expressing oocytes did not differ. In agreement with previous TEVC recordings of 5-HT_3_AR signalling in oocytes [60-63], 5-HT induced current responses in WT m5-HT_3_AR-oocytes in a concentration-dependent manner, exhibiting EC_50_ (pEC_50_ ± S.E.M.) and nH ± S.E.M. values of 3.6 μM (5.44 ± 0.13) and 1.3 ± 0.3 at the receptor (n = 7, Fig. 2A). Interestingly, the concentration-response relationship displayed by 5-HT at the receptors in m5-HT_3_A/ZAC-oocytes was substantially (12-fold) left-shifted [EC_50_ (pEC_50_ ± S.E.M.): 0.30 μM (6.54 ± 0.05), n = 10] compared to that at WT m5-HT_3_AR, and the Hill slope displayed by the agonist at the chimera was higher (nH ± S.E.M.: 2.3 ± 0.6, n = 10) (Fig. 2A). Notably, at concentrations higher than 10 μM the amplitudes of 5-HT-induced currents through m5-HT_3_A/ZAC decreased in a concentration-dependent manner, which could be indicative of a channel block (Fig. 2A).

Since Zn^2+^ is known to act as an allosteric modulator of m5-HT_3_AR signalling [33,36,37,64], we compared the modulatory properties of Zn^2+^ at 5-HT EC_80_-mediated responses through the m5-HT_3_A/ZAC chimera and WT m5-HT_3_AR (Fig. 2B). In agreement with previous findings [33], Zn^2+^ potentiated the 5-HT-induced response through WT m5-HT_3_AR at low-micromolar concentrations and inhibited it at higher concentrations (Fig. 2B). In contrast to this biphasic profile, Zn^2+^ displayed a monophasic concentration-inhibition relationship at m5-HT_3_A/ZAC characterized by an IC_50_ value similar to that at WT m5-HT_3_AR (Fig. 2B).

Finally, we compared the current responses evoked by sustained application of saturating agonist concentrations at m5-HT_3_A/ZAC, WT m5-HT_3_AR and WT ZAC. Representative traces are given in Fig. 2C and average kinetic characteristics extracted from all recorded traces are given in Table 1. In agreement with our previous study of ZAC in oocytes [24], currents evoked by sub-saturating Zn^2+^ concentrations in WT ZAC-oocytes were characterized by slow and negligible degrees of desensitization, and even though sustained application of Zn^2+^ (10 mM) produced currents characterized by a pronounced decay component, the decay was slow and resulted in a substantial level of residual current after 4 min (21%) (Fig. 2C, Table 1). In concordance with findings in previous studies of 5-HT_3_ARS [60,63], the currents evoked through WT m5-HT_3_AR by sustained application by a saturating 5-HT concentration (100 μM) were characterized by relatively fast desensitization and complete return to baseline after 98 ± 12 s (mean ± S.E.M., n = 7) (Fig. 2C, Table 1). In contrast, the currents evoked by a saturating 5-HT concentration (3 μM) through m5-HT3A/ZAC were characterized by a substantially slower activation phase than observed for WT ZAC and WT m5-HT_3_AR and even slower decay and higher levels of residual current after 4 min (67%) than observed for WT ZAC (Fig. 2C, Table 1).

#### 3.1.3. Functional characterization of the ZAC/hα_1_-Gly chimera

**Fig. 3.**
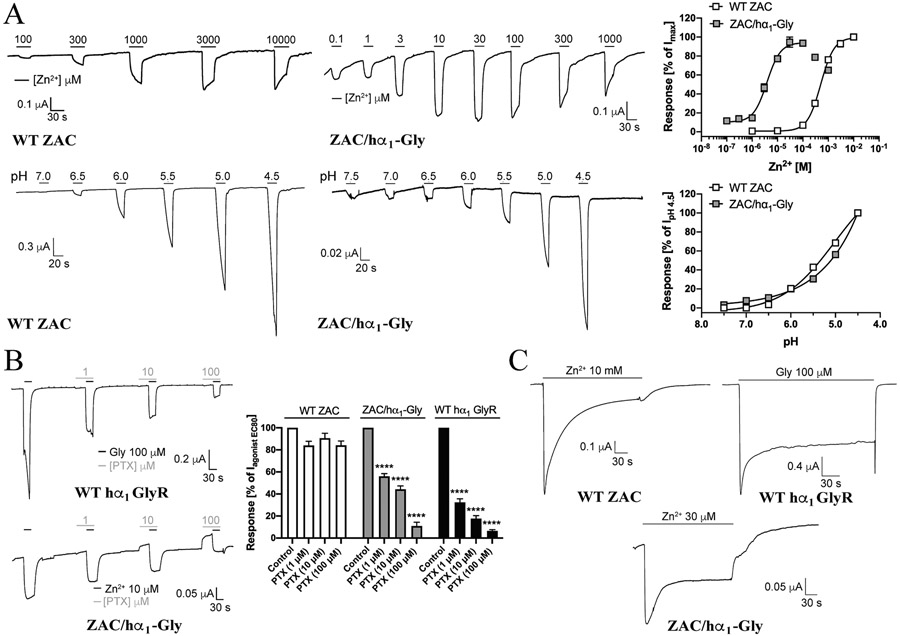
Functional properties exhibited by the chimeric ZAC/hα_1_-Gly receptor. **A.** Agonist properties displayed by Zn^+^ and H^+^ at ZAC/α_1_-Gly. Representative traces for Zn^2+^- and H^+^-evoked currents in WT ZAC- and ZAC/α_1_-Gly-expressing oocytes (*left*), and averaged concentration-response relationships displayed by Zn^2+^ and H^+^ at WT ZAC and ZAC/α_1_-Gly [means ± S.E.M., n = 8–10 (Zn^2+^) and n = 8–11 (H^+^)] (*right*). **B.** Antagonist properties displayed by picrotoxin (PTX) at ZAC/hα_1_-Gly. Representative traces for picrotoxin-mediated inhibition of Gly (EC_90_)-evoked currents in WT hα_1_ GlyR and Zn^2+^ (EC_90_)-evoked currents in ZAC/α_1_-Gly-expressing oocytes (*left*), and averaged concentration-inhibition relationships displayed by picrotoxin at WT hα_1_ GlyR, WT ZAC and ZAC/hα_1_-Gly (means ± S.E.M., n = 9–12) (*right*). Zn^2+^ (1 mM), Zn^2+^ (10 μM) and Gly (100 μM) were used for WT ZAC, ZAC/α_1_-Gly and WT hα_1_ GlyR, respectively. **C.** Representative traces of the currents evoked by 4 min-application of saturating agonist concentrations at WT hα_1_ GlyR, WT ZAC- and ZAC/α_1_-Gly-expressing oocytes. Zn^2+^ (10 mM), Zn^2+^ (30 μM) and Gly (100 μM) were used as agonist contrations for WT ZAC, ZAC/α_1_-Gly and WT hα_1_ GlyR, respectively. The trace for WT ZAC is the same as that shown in Fig. 2C.

Even though oocytes were injected with a 3-fold higher quantity of cRNA for ZAC/hα_1_-Gly than for WT ZAC and WT hα_1_ GlyR, the current amplitudes evoked by saturating concentrations of Zn^2+^ in ZAC/hα_1_-Gly-oocytes (I_max_: 0.24 ± 0.03 μA, n = 11) were somewhat smaller than those in WT ZAC-oocytes (I_max_: 0.58 ± 0.10 μA, n = 19) and substantially smaller those evoked by Gly in WT hα_1_ GlyR-oocytes (I_max_: 2.02 ± 0.24 μA, n = 5) (Fig. 1C). Zn^2+^ mediated currents in WT ZAC-expressing oocytes in a concentration-dependent manner, displaying an EC_50_ (pEC_50_ ± S.E.M.) value of 500 μM (3.30 ± 0.04, n = 8) (Fig. 3A). Strikingly, the concentration-response relationship determined for Zn^2+^ at ZAC/hα_1_-Gly was almost 100-fold left-shifted compared to that at WT ZAC, with Zn^2+^ displaying an EC_50_ (pEC_50_ ± S.E.M.) value of 3.8 μM (5.36 ± 0.09, n = 10) at the chimera (Fig. 3A). The Hill slopes of the fitted curves for the metal ion at the two receptors were similar [nH ± S. E.M.: 1.5 ± 0.2, n = 8 (WT ZAC); 1.6 ± 0.4, n = 10 (ZAC/hα_1_-Gly)]. Notably, at concentrations higher than 100 μM the amplitudes of Zn^2+^-evoked currents through ZAC/hα_1_-Gly decreased in a concentration-dependent manner, which could be indicative of a channel block (Fig. 3A). Protons also mediated concentration-dependent responses in WT ZAC- and ZAC/hα_1_-Gly-expressing oocytes (Fig. 3A). Since the H^+^ concentration-response relationships were not completed within the tested concentration range, EC_50_ (pH_50_) values at the two receptors could not be determined, but assessed from the pH values evoking significant currents through WT ZAC and ZAC/hα_1_-Gly, the agonist potencies displayed by H^+^ at the two receptors did not appear to differ substantially (Fig. 3A).

Next, we characterized the functional properties of picrotoxin (PTX), a promiscuous channel blocker characterized by substantially higher inhibitory potencies at anion-selective than at cation-selective CLRs, at ZAC/hα_1_-Gly and its two parent receptors. In agreement with the literature [31,65,66], PTX mediated concentration-dependent inhibition of Gly EC_90_-elicited responses through WT hα_1_ GlyR, exhibiting an estimated IC50 value slightly below 1 μM (Fig. 3B). In contrast, application of PTX at concentrations up to 100 μM did not inhibit Zn^2+^ EC_90_-mediated WT ZAC signalling significantly (Fig. 3B). PTX inhibited Zn^2+^ EC_90_-induced currents in ZAC/hα_1_-Gly-oocytes in a concentration-dependent manner, displaying an estimated IC_50_ value slightly above 1 μM at the chimera (Fig. 3B). Interestingly, the 30-sec preincubation of PTX was observed to result in substantial outward currents in the ZAC/hα_1_-Gly-oocytes, indicating a significant level of spontaneous activity in this channel. In contrast, no such outward currents were observed during the preincubation with PTX at WT hα_1_ GlyR (Fig. 3B).

Finally, we compared the profiles of the current responses evoked by sustained agonist application at WT ZAC, WT hα_1_ GlyR and ZAC/hα_1_-Gly (Fig. 3C, Table 1). As also shown in Fig. 2C, sustained application of Zn^2+^ (10 mM) at WT ZAC-expressing oocytes produced a current response characterized by slow decay and significant residual current (Fig. 3C, Table 1). In agreement with previous studies [10,15,67,68], the current evoked by 4 min application of Gly (100 μM) through WT hα_1_ GlyR was characterized by a fast activation phase followed by an initial decay phase yielding a plateau current that ultimately led to a considerable level of residual current after 4 min (52%) (Fig. 3C, Table 1). Interestingly, the profile of the current evoked by sustained Zn^2+^ (30 μM)-application at ZAC/hα_1_-Gly was qualitative similar to the Gly-evoked current through WT hα_1_ GlyR. While the activation phase for ZAC/hα_1_-Gly was slower than that for hα_1_ GlyR, the peak current observed for the chimera also decayed into a plateau current that resulted in a substantial residual current after 4 min (56%) (Fig. 3C, Table 1).

#### 3.1.4. Construction and characterization of modified ECD/TMD-ICD chimeras

It is by no means a given that fusion of an ECD and a TMD-ICD from two different CLR subunits results in a subunit capable of forming a functional CLR complex, as the unnatural combinations of ECD (pre-M1, β1-β2, β6-β7/Cys-loop, β8-β9) and TMD (M2-M3 linker, C-terminus) regions in the ECD/TMD-ICD chimeras are not always able to engage in the interdomain cross-talk needed for signal transduction. In previous studies, exchanges of selected of these ECD/TMD interface regions between the two CLRs fused in the ECD/TMD-ICD chimera have been found to alter the signalling properties of the chimeric receptor substantially [56,57,59,69] and in some cases even to convert nonfunctional chimeras into functional ones [58,59]. Furthermore, the specific fusion point between ECD and TMD-ICD in the chimeric subunit has also been found to be of key importance for the ability of the constructed chimera to form functional receptor complexes [52,53]. In an attempt to obtain functional ZAC/m5-HT_3_A and hα_1_-Gly/ZAC chimeras and to probe the functional consequences arising from such modifications to the functional m5-HT_3_A/ZAC and ZAC/hα_1_-Gly chimeras, we pursued both of these strategies.

**Fig. 4.**
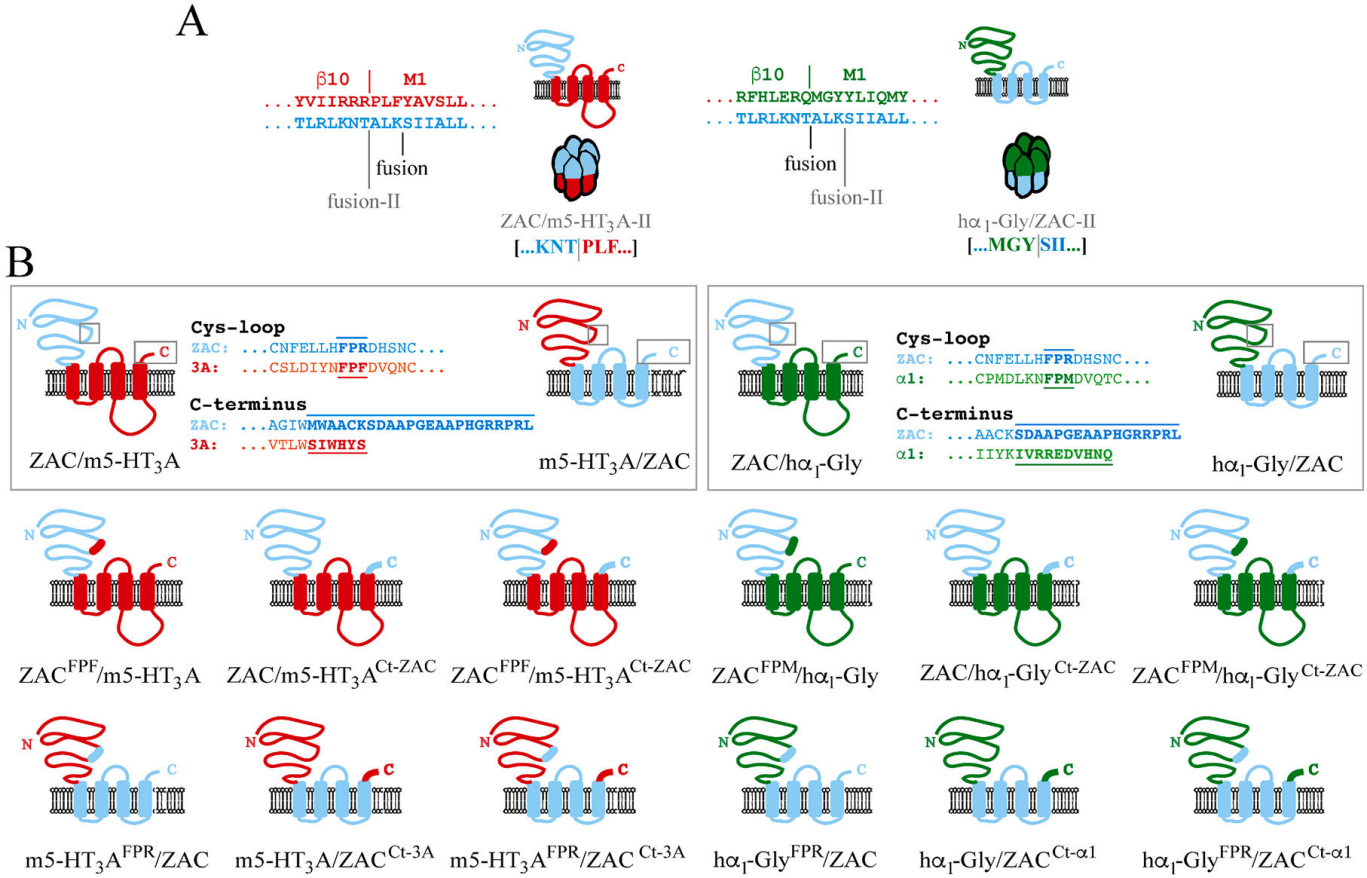
The alternative ZAC/m5-HT_3_A-II and hα_1_-Gly/ZAC-II chimeras and the ECD/TMD-ICD chimeras with Cys-loop and/or C-terminus modifications. **A.** Topologies of the chimeric ZAC/m5-HT_3_A-II and hα_1_-Gly/ZAC-II subunits and illustration of the pentameric complexes assembled from them. The amino acid sequences of the ECD-into-TMD sequences in the three WT subunit proteins are given, and the alternative fusion points in ZAC/m5-HT_3_A-II and hα_1_-Gly/ZAC-II (“fusion-II”) compared to those in the original ZAC/m5-HT_3_A and hα_1_-Gly/ZAC chimeras (“fusion”) are indicated. **B.** Schematic outline of the modifications made to the PFX-motif in the Cys-loop and in the C-terminal in the ECD-parts and the TMD/ICD-parts of the ZAC/m5-HT_3_A, m5-HT_3_A/ZAC, ZAC/hα_1_-Gly and hα_1_-Gly/ZAC chimeras.

As outlined above, the positions of the ECD/TMD fusion points in the ZAC/hα_1_-Gly and hα_1_-Gly/ZAC pair and in the ZAC/m5-HT_3_A and m5-HT_3_A/ZAC pair of chimeras differed, and only one chimera from each pair turned out to be functional (Fig. 1A). This prompted us to construct alternative versions of ZAC/m5-HT_3_A and hα_1_-Gly/ZAC in which the fusion points corresponded to those in ZAC/hα_1_-Gly and m5-HT_3_A/ZAC, respectively (termed ZAC/m5-HT_3_A-II and hα_1_-Gly/ZAC-II, Fig. 4A). However, just as the original ZAC/m5-HT_3_A and hα_1_-Gly/ZAC chimeras both of these new chimeras were non-functional, as Zn^2+^ (10 mM) and H^+^ (pH 4.0) did not evoke significant currents in ZAC/m5-HT_3_A-II-oocytes, and Gly (1 mM) did not induce significant responses in hα_1_-Gly/ZAC-II-oocytes (data not shown).

To probe the effects of exchanges of regions involved in the ECD/TMD cross-talk between the two CLRs fused in a chimera on its functionality, we constructed 12 additional chimeras, three for each of the four original chimeras (Fig. 1A). Inspired by the similar modifications that have been introduced in a functional GLIC/hα_1_-Gly chimera [57], we swapped one or both of two specific regions: the three-residue FPX (Phe-Pro-X) motif in the Cys-loop of the ECD (FPR, FPF and FMP in ZAC, m5-HT3A and hα_1_ GlyR, respectively), which is known to protude into proximity of the M2-M3 linker during the ECD/TMD cross-talk [10,12], and the extracellular C-terminus of the TMD, which differs substantially in both amino acid sequence and length between ZAC and the two classical CLRs combined with it in the chimeras (Fig. 4B). When these 12 modified chimeras were tested for functionality, only two of them were found to be functional. The concentration-response relationships exhibited by 5-HT at m5-HT_3_A^FPR^/ZAC and and by Zn^2+^ at ZAC^FPM^/hα_1_-Gly were not significantly different from those displayed by the agonists at m5-HT_3_A/ZAC and ZAC/hα_1_-Gly, respectively (data not shown). Furthermore, introduction of the FPF motif from m5-HT3A into the ECD of ZAC/m5-HT_3_A (ZAC^FPF^/m5-HT_3_A) and the FPR motif from ZAC into the ECD of hα_1_-Gly/ZAC (hα_1_-Gly^FPR^/ZAC) did not convert these nonfunctional chimeras into functional ones (data not shown). Finally, not only did the introduction of the C-terminus from the ECD-contributing CLR into the non-functional ZAC/m5-HT_3_A and hα_1_-Gly/ZAC chimeras not produce functional receptors (ZAC/m5-HT_3_A^Ct-ZAC^ and hα_1_-Gly/ZAC^Ct-alpha1^), the C-terminus swap was found to eliminate the functionality of m5-HT_3_A/ZAC and ZAC/hα_1_-Gly (m5-HT_3_A/ZAC^Ct-3A^ and ZAC/hα_1_-Gly^Ct-ZAC^) (data not shown). In light of this, it was not surprising that the four chimeras comprising exchanges of both the FPX motif and the C-terminus also were non-functional (data not shown). Given these findings, we did not pursue further experiments with these modified chimeras.

### 3.2. Search for the Zn^2+^ binding site(s) in ZAC

Collectively, the robust Zn^2+^- and H^+^-evoked currents through ZAC/hα_1_-Gly and the contrasting inability of the two ZAC agonists to evoke significant currents through the functionally expressed m5-HT_3_A/ZAC chimera strongly suggest that both the metal ion and protons mediate their ZAC activation predominantly through its ECD. This prompted us to search for the location of the putative Zn^2+^ binding site(s) in this domain, a search guided by the detailed insight into the structural requirements for Zn^2+^ binding to proteins gained from a plethora of crystal structures of enzymes and other proteins in complex with the metal ion published over the years [70-74]. Zn^2+^ binding to proteins is predominantly established through interactions with imidazole rings of His residues and thiol groups of Cys residues, but the metal ion also often forms ionic interactions with the carboxylate groups of Glu and Asp in these Zn^2+^/protein co-structures. Zn^2+^ binding is typically established via tetrahedral coordination to four interaction partners, be it direct interactions with the side chains of His, Cys, Glu or Asp residues in the protein or coordination to these via intermediate water molecules, with the typical interaction distances between Zn^2+^ and its binding partners being 2.5–3.0 Å [70-74].

**Fig. 5.**
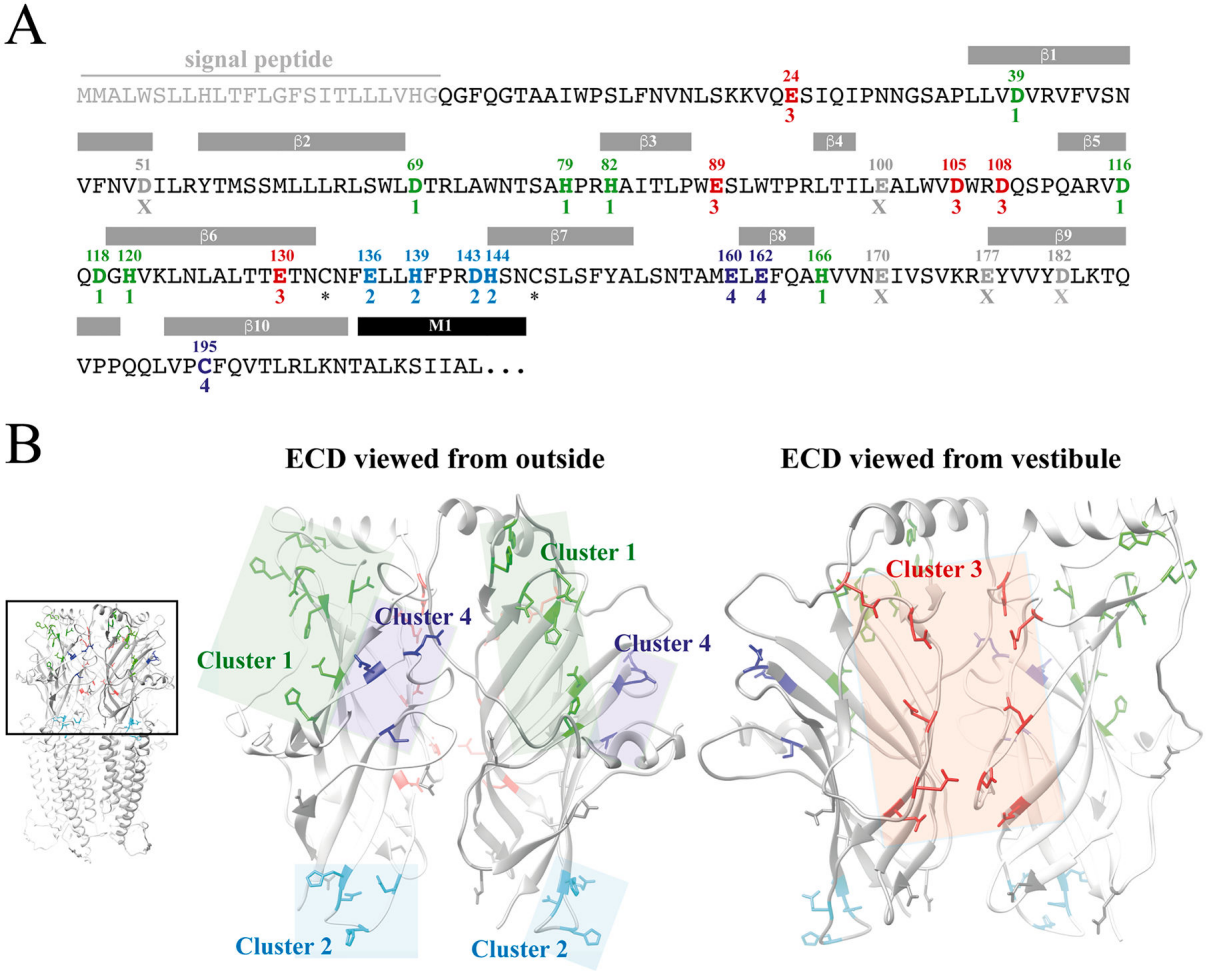
Candidate Zn^2+^-binding residues in the extracellular domain of ZAC. **A.** Amino acid sequence of the ZAC ECD. The indicated signal peptide, the β-sheet β1-β10 and the M1 α-helix segments in ZAC are predicted based on amino acid sequence aligment of the ZAC and m5-HT3A subunits and these segments in the m5-HT_3_AR cryo-EM structure (PDB ID: 6HIN) [11]. The 25 candidate Zn^2+^-binding residues in the ZAC ECD are given in bold with their residue numbers above. The candidate Zn^2+^-binding residues in the four defined clusters are given (Cluster 1: green; Cluster 2: cyan; Cluster 3: red; Cluster 4: dark-blue), with the five candidate residues not included in a cluster given in grey (“X”), and the two cysteines forming the Cys-loop are indicated with asterisks. **B.** Homology model of ZAC based on the cryo-EM structure of m5-HT_3_AR (PDB ID: 6HIN). The pentameric ZAC complex (*left*) and the ECD for two neighbouring subunits in the ZAC complex viewed from the outside (*middle*) and from the vestibule (*right*). The candidate Zn^2+^-binding residues in this domain defined as Cluster 1 (green), Cluster 2 (cyan), Cluster 3 (red) and Cluster 4 (dark-blue) are indicated in the ECD dimer, with the five candidate residues not included in a cluster shown in grey.

The ZAC ECD contains a total of 25 candidate Zn^2+^-binding residues: 6 His, 9 Glu and 9 Asp residues and a single Cys residue besides the two cysteines forming the Cys-loop in the subunit (Fig. 5A). Based on a homology model of ZAC [24], we identified four clusters (Clusters 1, 2, 3 and 4) of candidate Zn^2+^-binding residues in the ECD, which each comprised a sufficient number of residues characterized by interresidual distances and spatial orientations that could be envisioned to accommodate Zn^2+^ coordination (Fig. 5B). However, it should be noted that the grouping of these candidate Zn^2+^-binding residues in clusters allowed for inter-residual distances between the thiol (Cys), imidazole (His) and carboxylate (Glu, Asp) moieties of the candidate residues to be larger than the optimal distances of 6–8 Å (enabling Zn^2+^-residue distances of 2.5–3.0 Å) and also for inter-residual geometries to be less than ideal for Zn^2+^ coordination (Fig. 5B, Table 2). This was done in part because of the unrefined state of our ZAC homology model and in part to take into account possible induced-fit binding of the metal ion to its coordinating residues. Collectively, the four defined clusters comprised 20 of the 25 Zn^2+^-binding candidate residues in the ZAC ECD, with the remaining five candidate residues being scattered across the domain with distances to other Zn^2+^-binding candidate residues (according to the ZAC homology model) that were deemed to be too high to accommodate metal ion coordination (Fig. 5A and B).

The putative involvement of candidate residues in Clusters 1, 2, 3 and 4 in Zn^2+^ binding to ZAC was investigated in by an elaborate alanine mutagenesis scanning. The functional expression levels of the mutants were assessed and compared to that displayed by WT ZAC in the oocytes by recording of the current amplitudes evoked by both Zn^2+^ (10 mM) and H^+^ (pH 4.0). The determined I_pH 4.0_ value was used in part as a control and in part to enable the distinction of ZAC mutants non-responsive to Zn^2+^ due to elimination of their ability to bind the metal ion from those where the mutation had eliminated cell surface expression and/or disrupted overall functionality of the receptor complex. As we do not have an assay enabling quantification of ZAC expression levels at the oocyte surface [24], ZAC mutants found to be non-responsive to both Zn^2+^ and H^+^ as agonists could either be surface-expressed non-functional mutants or mutants not expressed at the oocyte surface. In these relatively few cases, the putative importance of the residue(s) mutated in the non-responsive mutants for Zn^2+^-evoked ZAC gating were subsequently investigated in subsequent rounds of mutants.

As it will also be outlined below, the investigation was complicated by the agonist potency displayed by Zn^2+^ at WT ZAC in a couple of oocyte-batches being 5–10-fold higher (EC_50_ ~100 μM) than the “normal” agonist potency displayed by the metal ion at the receptor in the vast majority of oocytes in this work and in two other studies (EC_50_ ~0.5–1 mM) [24,75]. These differences in the Zn^2+^ concentration-response relationship appeared to be completely oocyte batch-dependent, as the EC_50_ values determined for the metal ion at different WT ZAC-expressing oocytes originating from the same batch were highly comparable. Reminiscent of this observation, we observed even more dramatic differences in the agonist profiles for H^+^ at ZAC expressed in oocytes from different batches in a recent study, but here the agonist potency displayed by Zn^2+^ was very stable across various oocyte batches [24]. The reason(s) for those oocyte-dependent “high-potency” and “low-potency” agonist profiles for H^+^ and for the differences in Zn^2+^ concentration-response relationships at WT ZAC observed in this work are presently unknown. In view of this and to enable a valid and reliable assessment of the impact of various mutations on Zn^2+^-evoked ZAC signalling, we thus performed parallel TEVC recordings at WT ZAC and ZAC mutants expressed in oocytes from the same batches to facilitate a direct comparison of the concentration-response relationships exhibited by Zn^2+^ at the receptors.

#### 3.2.1. Cluster 1

**Fig. 6.**
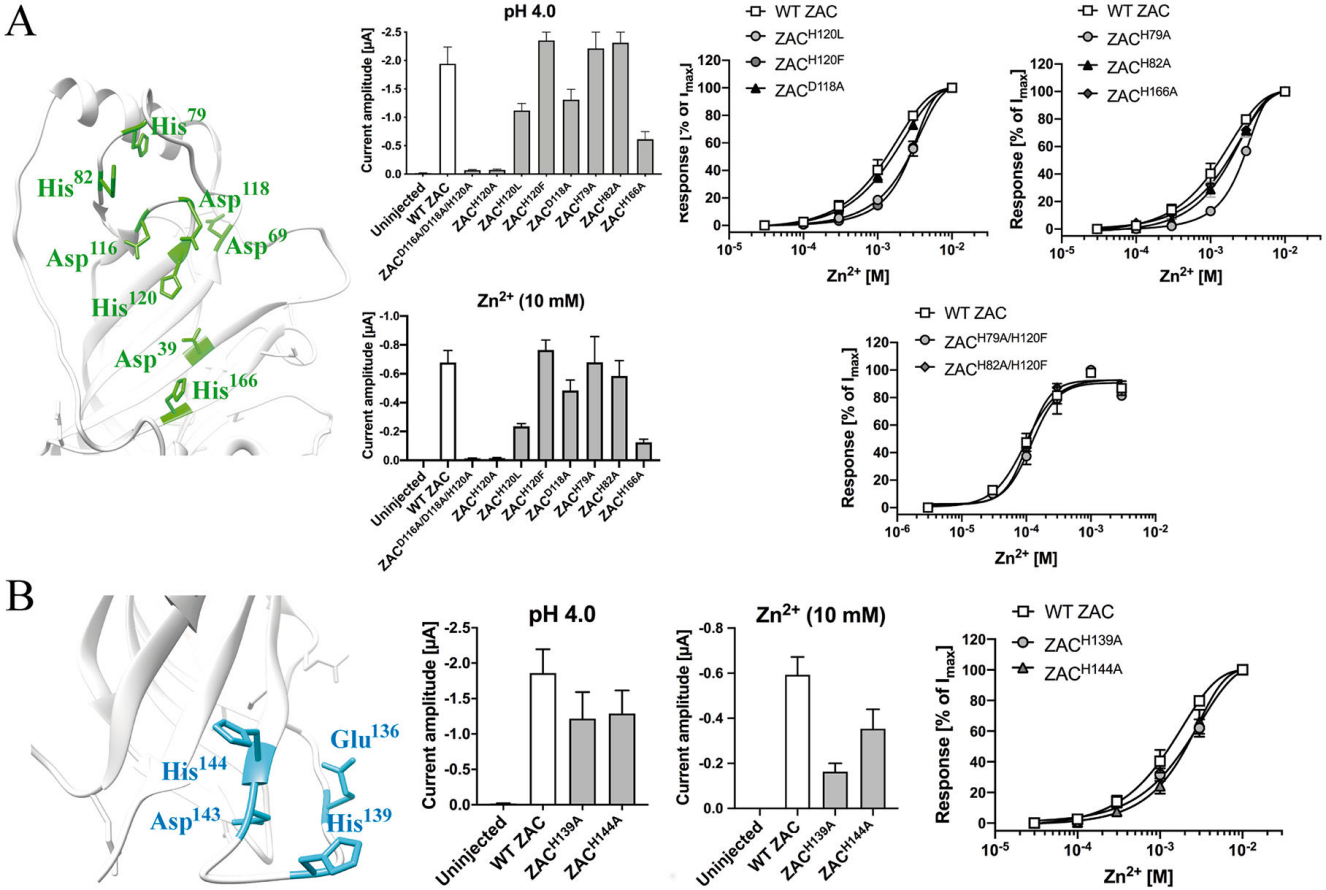
Probing the importance of candidate Zn^2+^-binding residues in Clusters 1 and 2 of the ZAC ECD for Zn^2+^-mediated ZAC activation. **A.** Cluster 1. *Left:* Cluster 1 residues (in green, detail of ZAC homology model). *Middle:* Averaged I_pH 4.0_ and I_10 mM Zn2+_ values recorded from oocytes expressing WT ZAC and various ZAC mutants [means ± S.E.M., H^+^: n = 5–8 (mutants), n = 14 (WT); Zn^2+^: n = 6–8 (mutants), n = 16 (WT)]. *Right:* Averaged concentration-response relationships displayed by Zn^2+^ at oocytes expressing WT ZAC and various ZAC mutants [Top graphs: means ± S.E.M., n = 6–8 (mutants), n = 14 (WT). Bottom graph: means ± S.E.M., n = 6–8]. **B.** Cluster 2. *Left:* Cluster 2 residues (in cyan, detail of ZAC homology model). *Middle:* Averaged I_pH 4.0_ and I_10 mM Zn2+_ values recorded from oocytes expressing WT ZAC and various ZAC mutants [means ± S.E.M., H^+^: n = 6–8; Zn^2+^: n = 6–7]. *Right:* Averaged concentration-response relationships displayed by Zn^2+^ at oocytes expressing WT ZAC, ZAC^H139A^ and ZAC^H144A^ [means ± S.E.M., n = 7–8 (mutants), n = 14 (WT)].

This intra-molecular cluster comprises two subclusters of residues, each of which potentially could form a Zn^2+^ binding site (Fig. 5B and 6A, *left*). Both subclusters comprise the Asp^116^, Asp^118^ and His^120^ residues in the β5-β6 loop, which are located 5–6 Å from each other (Table 2). This triad of residues is supplemented with His^79^ and His^82^ in the β2-β3 loop positioned above the β5-β6 loop in one subcluster (inter-residual distances of 8–13 Å) and with Asp^39^ (β1), Asp^69^ (β2-β3 loop) and His^166^ (β8) located on the opposite site of the β5-β6 loop in the other subcluster (inter-residual distances of 5–19 Å) (Table 2, Fig. 6A, *left*).

Alanine substitutions of all three residues shared by the two subclusters resulted in a receptor that was close to non-responsive to Zn^2+^ and H^+^, with I_10 mM Zn2+_ and I_pH 4.0_ ranges recorded from ZAC^D116A/DH8A/H210A^-expressing oocytes being 10–30 nA and 40–100 nA, respectively (Fig. 6A, *middle*). A single alanine substitution of His^120^ in ZAC had a similar detrimental impact on Zn^2+^- and H^+^-evoked current amplitudes, with I_10 mM Zn2+_ and I_pH 4.0_ values from ZAC^H210A^-oocytes being 5–30 nA and 30–110 nA, respectively, Fig. 6A, *middle*). These minute current amplitudes made it impossible to determine the concentration-relationships for Zn^2+^ at the two mutants. In contrast to the negligible functionality of ZAC^H120A^, substitution of His^120^ for a Leu or a Phe residue yielded receptors characterized by robust functional expression, and interestingly Zn^2+^ exhibited slightly lower agonist potency at these mutants, with fitted EC_50_ values at both ZAC^H120L^ and ZAC^H120F^ being ~3-fold higher than that at WT ZAC (Fig. 6A). Substitution of Asp^118^ for Ala was not detrimental to functional expression of ZAC, and the agonist properties displayed by Zn^2+^ at ZAC^D118A^ did not differ substantially from those at the WT receptor (Fig. 6A).

The putative involvement of three of the five other Cluster 1 residues in Zn^2+^ binding was probed by Ala substitutions of the His^79^, His^82^ and His^166^ residues. Whereas Zn^2+^ exhibited WT-like agonist properties at ZAC^H82A^ and ZAC^H166A^, the fitted EC_50_ value for the concentration-response relationship displayed by the metal ion at ZAC^H79A^ was 2.4-fold higher than that at WT ZAC in parallel recordings (Fig. 6A, *right*). The modestly reduced agonist potencies displayed by Zn^2+^ at ZAC^H79A^ and ZAC^H120F^ prompted us to characterize the agonist properties of the metal ion at the double mutant combining these two mutations and at the triple ZAC^H79A/H82A/H120F^ mutant. In the oocytes used for these experiments, Zn^2+^ consistently displayed higher agonist potency at WT ZAC (EC_50_ ~100 μM, Fig. 6A *right*) than its potency at the receptor at the vast majority of oocyte batches in this work and in previous studies (EC50 ~0.5–1 mM) [24,75]. However, judging from the agonist properties exhibited by Zn^2+^ at the ZAC^H79A/H120F^, ZAC^H82A/H120F^ and ZAC^H79A/H82A/H120F^ mutants and at WT ZAC expressed in oocytes from the same batches in parallel TEVC recordings, the concomitant elimination of the imidazole ring systems in position 79, 82 and 120 did not appear to impair the ability of Zn^2+^ to elicit ZAC signalling (Fig. 6A, *right*).

#### 3.2.2. Cluster 2

This intra-molecular cluster consists of the candidate Zn^2+^-binding residues Glu^136^, His^139^, Asp^143^ and His^144^ in the Cys-loop (β6/β7) of ZAC (Fig. 6B, *left*). According to the ZAC homology model, the distances between the carboxylate and imidazole functionalities of these four residues are between 6.8 and 8.5 Å and thus well within a range where Zn^2+^ coordination between them seems feasible (Table 2). That said, metal ion coordination to residues in the Cys-loop does seem unlikely given the key role of the loop in the ECD/TMD cross-talk and its need to be flexible and to move in order to fulfil this role [10-16]. On the other hand, the proximity of the Cys-loop to the M2-M3 linker and other extracellular TMD regions during the signal transduction through the receptor could potentially lead to the formation of an inter-domain Zn^2+^ binding site composed by residues in the loop and residues from the TMD.

Alanine substitutions of all four candidate Zn^2+^-binding residues in the Cys-loop rendered ZAC non-responsive to both Zn^2+^ (10 mM) and H^+^ (pH 4.0) in oocytes (ZAC^E136A/H139A/D143A/H144A^, data not shown). We did not investigate the underlying reasons for this lack of functionality, but it is most likely attributable to disruption of the ECD/TMD cross-talk due to the dramatic modifications introduced in the Cys-loop. Asp^143^ in ZAC is completely conserved as an Asp residue in all mammalian CLRs, and the carboxylate group of this residue has been shown to form key ionic interactions in the ECD/TMD interface and to be of key importance for the signal transduction [12,17,76]. In light of the conserved essential role of this Asp residue for CLR gating, we did not subject Asp^143^ in ZAC to mutagenesis but instead focused on the two histidines in the Cys-loop. Zn^2+^-evoked currents through ZAC^H139A^ and ZAC^H144A^ were characterized by lower maximal amplitudes (I_10 mM Zn2+_) than those in WT ZAC-oocytes, but the functional properties of the metal ion as a ZAC agonist were not altered significantly by either of the two mutations (Fig. 6B).

#### 3.2.3. Cluster 3

**Fig. 7.**
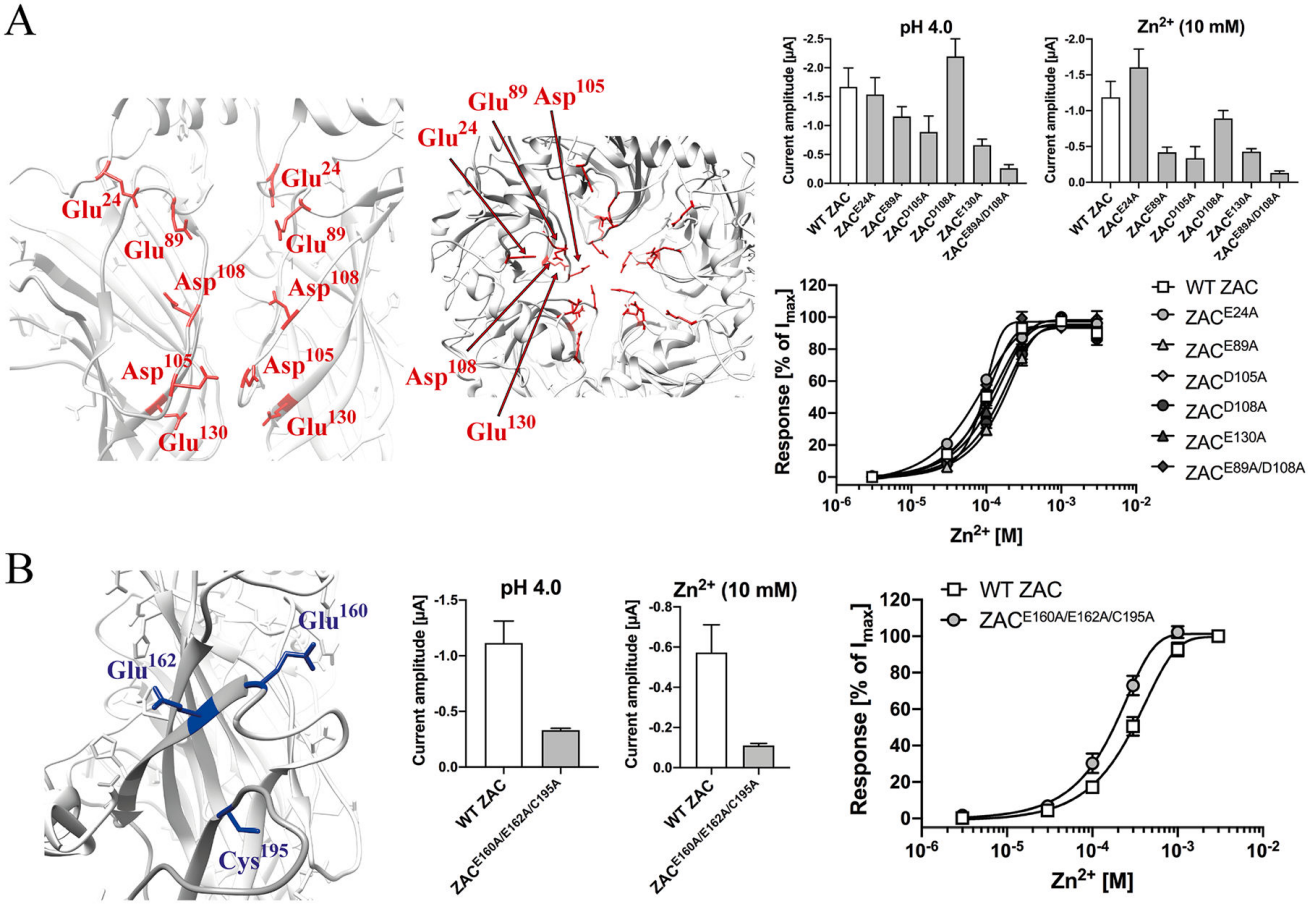
Probing the importance of candidate Zn^2+^-binding residues in Clusters 3 and 4 of the ZAC ECD for Zn^2+^-mediated ZAC activation. *A.* Cluster 3. *Left:* Cluster 3 residues (in red, detail of ZAC homology model). *Right, top:* Averaged I_pH 4.0_ and I_10 mM Zn2+_ values recorded from oocytes expressing WT ZAC and various ZAC mutants [means ± S.E.M., n = 5–8]. *Right, bottom:* Averaged concentration-response relationships displayed by Zn^2+^ at oocytes expressing WT ZAC and various ZAC mutants [means ± S.E.M., n = 5–8 (mutants), n = 12 (WT)]. **B.** Cluster 4. *Left:* Cluster 4 residues (in dark-blue, detail of ZAC homology model). *Middle:* Averaged I_pH 4.0_ and I_10 mM Zn2+_ values recorded from oocytes expressing WT ZAC and various ZAC mutants [means ± S.E.M., n = 5–6]. *Right:* Averaged concentration-response relationships displayed by Zn^2+^ at oocytes expressing WT ZAC and ZAC^E160A/E162A/C195^ [means ± S.E.M., n = 5–6]. WT ZAC- and ZAC^E160A/E162A/C195^-oocytes were injected with 1.84 ng and 3.68 ng cRNA, respectively.

This inter-molecular cluster is composed by five acidic residues lining the vestibule of the ECD in the ZAC subunit: Glu^24^ (loop between the *N*-terminal α-helix and β1), Glu^89^ (β3-β4 loop), Asp^105^ and Asp^108^ (β4-β5 loop) and Glu^130^ (β6) (Fig. 5A and 7A, *left*). Several intra-molecular inter-residual distances (i.e. between residues in the same subunit) as well as inter-molecular inter-residual distances (i.e. between residues in two neighboring subunits) in this cluster are well within ranges that would make Zn^2+^ coordination between them feasible, keeping in mind the possibility of an induced-fit binding mechanism and the ability of water molecules to bridge between Zn^2+^ and its interaction partners [70-74] (Table 2, Fig. 7A, *left*). The cluster can be divided into two subsections each composed by Asp^108^ and two of the other acidic residues: an upper subsection composed by Asp^108^, Glu^24^ and Glu^89^ (intra-molecular and inter-molecular distances: 6.7–11.3 Å and 10.5–20.4 Å, respectively) and a lower subsection composed by Asp^108^, Asp^105^ and Glu^130^ (intra-molecular and inter-molecular distances: 8.0–14.8 Å and 5.6–17.8 Å, respectively) (Table 2, Fig. 7A, *left*).

Substitution of all five of these residues in Cluster 3 for alanines resulted in a mutant receptor (ZAC^E24A/E89A/D105A/D108A/E130A^) that was non-responsive to both H^+^ (pH 4.0) and Zn^2+^ (10 mM) (data not shown). Thus, we next characterized the functional properties of Zn^2+^ at ZAC^E24A^, ZAC^E89A^, ZAC^D105A^, ZAC^D108A^ and ZAC^E130A^, since both Zn^2+^ (10 mM) and H^+^ (pH 4.0) elicited robust currents in the oocytes expressing these five mutants (Fig. 7A, *middle*). Analogously to the higher agonist potency observed for Zn^2+^ at WT ZAC in some of the TEVC recordings performed for the Cluster 1 investigations (outlined in section 3.2.1), the concentration-response curves determined for Zn^2+^ at WT ZAC in the oocytes used in these recordings were consistently left-shifted compared to those typically observed for the metal ion, with Zn^2+^ displaying an averaged EC_50_ value of 98 μM (pEC_50_ ± S.E.M.: 4.01 ± 0.03, n = 12) at the WT receptor in these recordings (Fig. 7A, *right*). The concentration-response relationships determined for Zn^2+^ at all five point-mutated receptors did not differ significantly from that at WT ZAC, as the averaged EC_50_ values for the mutants ranged from 71 μM (pEC_50_ ± S.E.M.: 4.15 ± 0.04, n = 5) for ZAC^E24A^ to 160 μM (pEC_50_ ± S.E.M.: 3.79 ± 0.07, n = 7) for ZAC^E89A^ (Fig. 7A, *right*). In other mutants, we combined alanine substitutions of two of the five residues. ZAC^D105A/D108A^- and ZAC^D105A/E130A^-expressing oocytes were found to be non-responsive to both pH 4.0 and 10 mM Zn^2+^ (data not shown). ZAC^E89A/D105A^ was functionally expressed in the oocytes albeit at significantly lower levels than WT ZAC, and the Zn^2+^ concentration-response relationship determined at this mutant was very similar to that at WT ZAC (Fig. 7A, *right*).

#### 3.2.4. Cluster 4

According to the ZAC homology model, the carboxylate groups of Glu^160^ (β7/β8 loop) and Glu^162^ (β8) and the thiol group in Cys^195^ (β10) in Cluster 4 are positioned 12–15 Å from earch other (Table 2, Fig. 7B, *left*). If the homology model is representative when it comes to this region, Zn^2+^ coordination between these three residues would thus require an induced-fit mechanism with substantial movement of the residues towards each other. The functional expression level of the ZAC^E160A/E162A/C195A^ mutant in oocytes was found to be significantly lower than that of WT ZAC (Fig. 7B, *right*), but the concentration-response relationship exhibited by Zn^2+^ at the triple mutant (EC_50_: 180 μM; pEC_50_ ± S.E.M.: 3.75 ± 0.05, n = 6) did not differ substantially from that at WT ZAC (EC_50_: 310 μM; pEC_50_ ± S.E.M.: 3.51 ± 0.05, n = 5) in parallel recordings (Fig. 7B, *right*).

### 3.3. Investigation of the functional importance of the Leu9′ residue in ZAC

In the final part of the study, we probed the importance of the Leu^246^ residue in the M2 α-helix in ZAC, hereafter termed Leu9′, for ZAC function. This Leu residue is highly conserved throughout the members of the CLR superfamily, and the “leucine ring” in the ion channel formed by molecular interactions between the sidechains of the five Leu9’ residues in the pentameric complex constitutes the gate in the resting CLR conformation [12,16]. Even conservative mutations of Leu9’ in classical CLRs have been shown to dramatically alter the channel properties, giving rise to slower desensitization kinetics, substantial levels of constitutive activity and left-shifted agonist concentration-response relationships at the receptors [77-81].

**Fig. 8.**
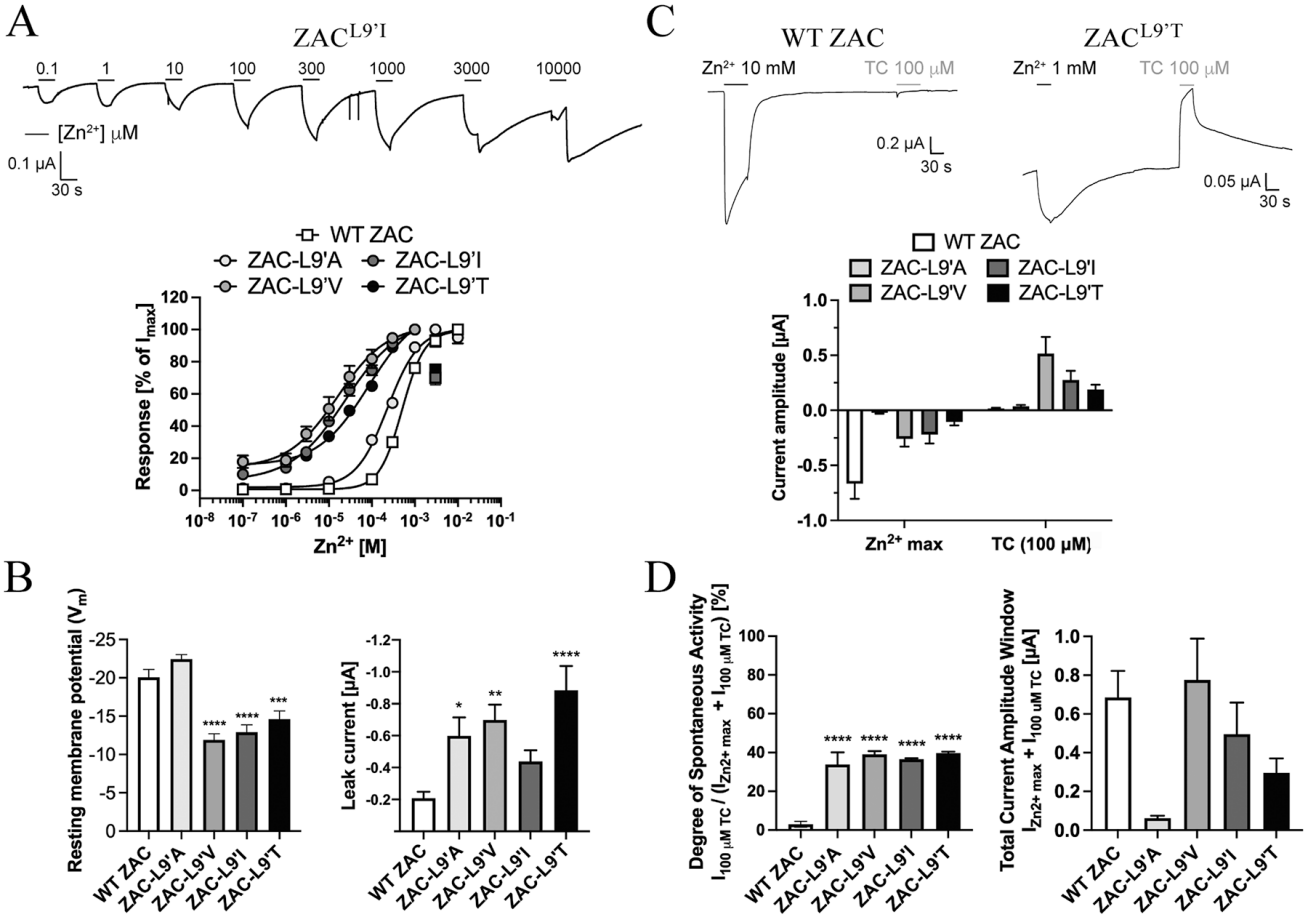
Functional importance of the Leu9′ residue in ZAC. **A.** Representative traces for Zn^2+^-evoked currents in ZAC^L9′I^-expressing oocytes (*top*), and averaged concentration-response relationships exhibited by Zn^2+^ at WT ZAC, ZAC^L9′A^, ZAC^L9′V^, ZAC^L9′I^ and ZAC^L9′T^ (means ± S.E.M., n = 6–8) (*bottom*). **B.** Resting membrane potentials (*left*) and leak currents (*right*) recorded from oocytes expressing WT ZAC, ZAC^L9′A^, ZAC^L9′T^, ZAC^L9′V^ and ZAC^L9′I^. Data are given as mean ± S.E.M. values (n = 40–60). **C.** Representative traces for Zn^2+^- and TC (100 μM)-evoked currents in WT ZAC- and ZAC^L9′I^-expressing oocytes (*top*), and averaged current amplitudes evoked by a saturating concentration of Zn^2+^ and by TC (100 μM) in WT ZAC-, ZAC^L9′A^-, ZAC^L9′V^-, ZAC^L9′I^-, and ZAC^L9′T^-oocytes (means ± S.E.M., n = 6–8) (*bottom*). 10 mM Zn^2+^ were used for WT ZAC and 1 mM Zn^2+^ were used for ZAC^L9′A^, ZAC^L9′V^, ZAC^L9′I^ and ZAC^L9′T^. **D.** Degrees of spontaneous activity [defined as: I_100 uM TC_/ (I_Zn2+ max_ + I_100 uM TC_)] (*left*) and total current amplitude windows (defined as: I_Zn2+ max_ + I_100 uM TC_) (*right*) exhibited by WT ZAC, ZAC^L9′A^, ZAC^L9′V^, ZAC^L9′I^ and ZAC^L9′T^ expressed in oocytes.

The putative importance of Leu9′ for ZAC functionality was investigated by comparing the functional properties exhibited by Zn^2+^ at four mutants comprising Ala, Val, Ile or Thr in this position with those displayed at WT ZAC. Zn^2+^ was found to induce significant current responses in a concentration-dependent manner through all four ZAC^L9′X^ mutants expressed in oocytes (exemplified for ZAC^L9′I^ in Fig. 8A, *top*). However, while Zn^2+^ exhibited largely comparable concentration-response relationships at ZAC^L9′A^ and at WT ZAC, displaying EC_50_ (pEC_50_ ± S.E.M.) and n_H_ ± S.E.M. values of 230 μM (3.64 ± 0.08) and 1.2 ± 0.2 at the mutant (n = 7), the metal ion displayed significantly higher agonist potencies and lower Hill slopes at the three other ZAC^L9′X^ mutants [EC_50_ (pEC_50_ ± S.E.M.) and n_H_ ± S.E.M.: ZAC^L9′V^: 13 μM (4.88 ± 0.22) and 0.7 ± 0.3 (n = 8); ZAC^L9′I^: 24 μM (4.62 ± 0.15) and 0.8 ± 0.2 (n = 8); ZAC^L9′T^: 66 μM (4.18 ± 0.34) and 0.7 ± 0.4 (n = 6)] (Fig. 8A, *bottom*). As will be addressed below, the apparent activation, deactivation and desensitization kinetics observed for Zn^2+^ at the four mutants were substantially slower than those at WT ZAC, and thus the Zn^2+^ concentration-response relationships for the four mutants extracted from the recordings had to be based on current responses that did not reach saturation within the 30 s application of Zn^2+^. However, it was also evident by visual comparison of the current responses mediated by various Zn^2+^ concentrations in ZAC^L9′V–^, ZAC^L9′I^-, ZAC^L9′T^- and WT ZAC-oocytes that the metal ion was a considerably more potent agonist at the mutants than at the WT receptor, and we propose that the EC_50_ values extracted from these data for the mutant receptors are reasonably representative.

The current amplitudes evoked by saturating Zn^2+^ concentrations (I_Zn2+_ max) in ZAC^L9′A^-, ZAC^L9′V^-, ZAC^L9′I^- and ZAC^L9′T^-expressing oocytes were substantially lower than those in WT ZAC-oocytes, in particular those in the ZAC^L9′A^-oocytes. Interestingly, however, the resting membrane potentials recorded in the ZAC^L9′V^-, ZAC^L9′I^- and ZAC^L9′T^-oocytes were significantly higher than those in WT ZAC-oocytes, and the leak currents recorded from mutant-expressing oocytes were also markedly higher than those in WT ZAC-oocytes (Fig. 8B). This prompted us to assess and compare the levels of spontaneous activity exhibited by WT ZAC and the four ZAC mutants by use of tubo-curarine (TC), a promiscuous antagonist of cation-selective CLRs, including ZAC [22-24]. Whereas application of TC (100 μM) at WT ZAC- and ZAC^L9′A^-expressing oocytes evoked small but significant outward currents (I_100 μM TC_), the outward currents induced by the antagonist in ZAC^L9′V^-, ZAC^L9′I^- and ZAC^L9′T^-oocytes were characterized by substantially higher amplitudes (Fig. 8C). The four mutant receptors exhibited very similar degrees of spontaneous activity [assessed by the I_TC 100 μM_/(I_100 μM TC_ + I_Zn2+ max_ ratio)], all of which that were substantially higher than the degree of spontaneous activity displayed by WT ZAC (Fig. 8D, *left*). Interestingly, the total current amplitude windows (assessed by the numeric sum of I_Zn2+ max_ and I_100 μM TC_) recorded in ZAC^L9′V^- and ZAC^L9′I^-oocytes did not differ significantly from that in WT ZAC-oocytes (Fig. 8D, *right*). Even though it should be noted that 2.5–3.2-fold higher quantities of ZAC^L9′X^ mutant cRNA than WT ZAC cRNA were injected into the oocytes used for these experiments, this suggests that the lower I_Zn2+ max_ values recorded from ZAC^L9′V^- and ZAC^L9′I^-oocytes are not rooted in substantially lower functional expression of these two mutants compared to WT ZAC, but rather reflects that the equilibria between conducting (active) and non-conducting (resting and/or desensitized) ZAC conformations have been changed by the introduced L9′V/L9′I mutation. Although similar changed equilibria between receptor conformations was observed for ZAC^L9′T^ and ZAC^L9′A^ mutants (Fig. 8D, *left*), the lower total current amplitude windows recorded from oocytes expressing these two ZAC^L9′X^ mutants suggest that the functional expression levels of these two mutants, in particular that of ZAC^L9′A^, also are significantly reduced compared to that of the WT receptor (Fig. 8D, *right*).

**Fig. 9.**
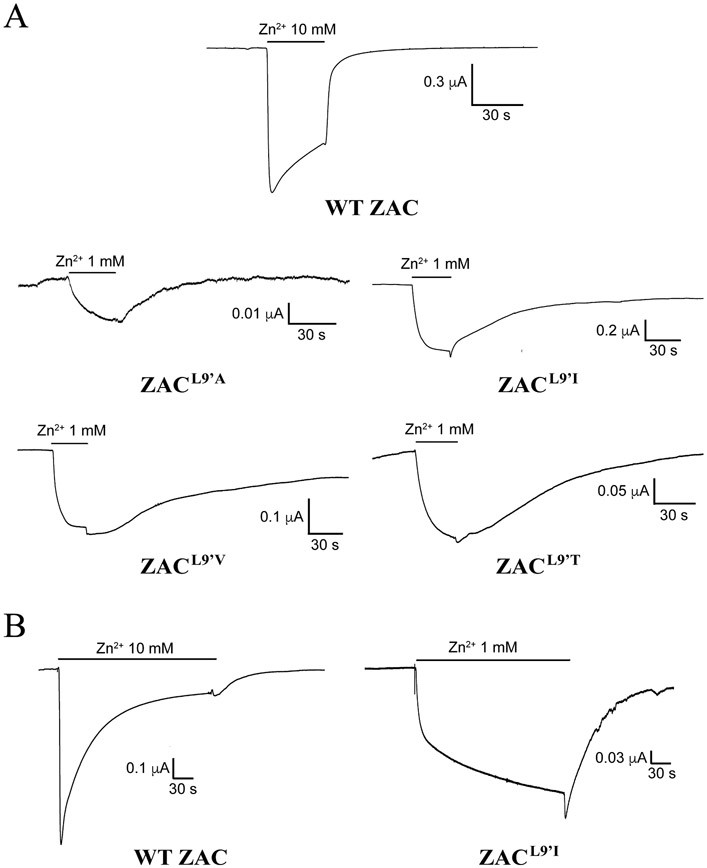
*Signalling characteristics exhibited by the* ZAC^L9′X^ *mutants*. **A.** Representative traces of currents evoked by saturating Zn^2+^ concentrations in WT ZAC-, ZAC^L9′A^-, ZAC^L9′V^-, ZAC^L9′I^- and ZAC^L9′T^-expressing oocytes. 10 mM Zn^2+^ was used for WT ZAC and 1 mM Zn^2+^ was used for the ZAC^L9′X^ mutants, respectively. **B.** Representative traces of currents evoked by sustained application of saturating Zn^2+^ concentrations in WT ZAC- and ZAC^L9′I^-expressing oocytes. 10 mM Zn^2+^ and 1 mM Zn^2+^ were used for WT ZAC and for ZAC^L9′I^, respectively.

It was noticeable already in the recordings delineating the Zn^2+^ concentration-response relationships that the channel properties displayed by the four ZAC^L9′X^ receptors were markedly distinct from those exhibited by WT ZAC. One should be cautious when extrapolating information about channel kinetics based on TEVC recordings from oocytes. Nevertheless, these differences were assessed further by comparing the current responses evoked by saturating Zn^2+^ concentrations at WT ZAC (10 mM Zn^2+^) and at the mutants (1 mM Zn^2+^) in the oocytes. In these recordings, the apparent activation kinetics observed for WT ZAC was considerably faster than those displayed by the ZAC^L9′X^ mutants, and the four mutants also deactivated much slower than the WT receptor (Fig. 9A). Furthermore, the profiles of the current responses evoked by sustained applications of saturating Zn^2+^ concentrations at WT ZAC and the four mutants revealed that the already slow desensitization kinetics of ZAC is decreased even further by the introduction of a L9′X mutation in the receptor (exemplified for ZAC^L9’I^ in Fig. 9B).

## 4. Discussion

The present work represents the first investigation into the molecular basis for the signal transduction through ZAC, a hitherto largely unexplored CLR. The findings offer the first glimpses into the mechanisms underlying ZAC signalling and the extent to which they resemble those underlying the signalling of the classical CLRs.

### 4.1. Signal transduction through ZAC investigated by ECD/TMD-ICD chimeras

**Fig. 10.**
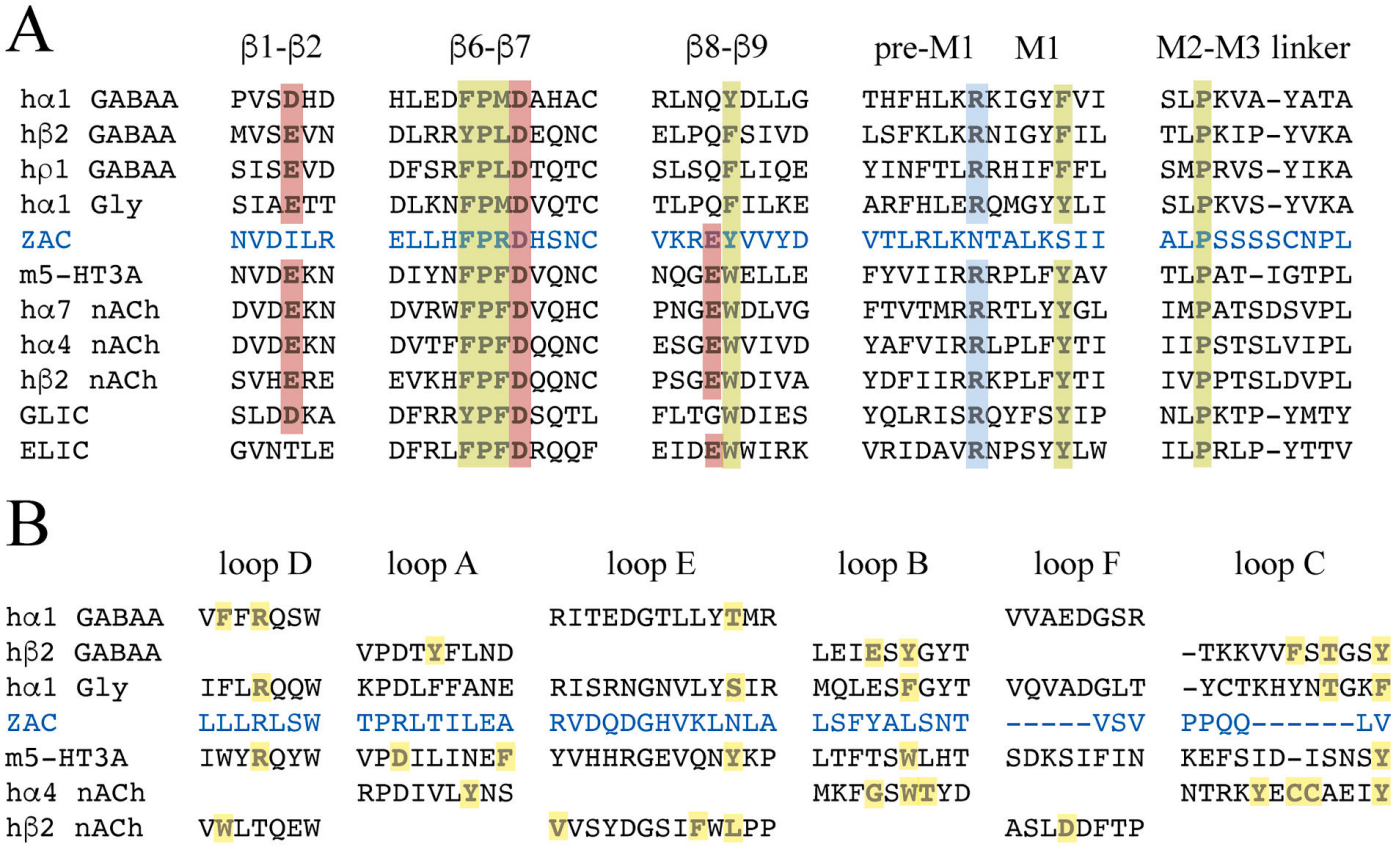
Key residues involved in signal transduction through the CLR. **A.** Residues involved in ECD/TMD cross-talk in the classical CLR. Alignment of the amino acid sequences of the β1-β2, β6-β7 (Cys-loop) and β8-β9 loops, the pre-M1/M1 segments and the M2-M3 linkers in ZAC, selected classical CLRs and the prokaryotic CLRs GLIC and ELIC. The conservation of key residues for the ECD/TMD cross-talk are indicated (negatively charged or charge-neutral, polar residues in red and positively charged residues blue, structural residues in green). **B.** Residues involved in orthosteric agonist binding to the classical CLR. Alignment of the amino acid sequences of loops A-F in ZAC and selected classical CLRs. The residues in the loops directly involved in orthosteric agonist binding to m5-HT_3_AR [12], hα_4_β_2_ nAChR [14], hα_1_β_2_γ*2* GABA_A_R [13] and hα_1_ GlyR [10] are indicated in bold and highlighted in yellow.

Although m5-HT_3_A/ZAC and ZAC/hα_1_-Gly are not the first functional chimeric receptors arising from fusions of ECDs and TMD-ICDs from distantly related receptors, their ability to translate agonist binding to the ECD into channel gating in the TMD is nevertheless remarkable. The atomic level insight into the signal transduction through classical CLRs offered by numerous recent high-resolution structures, including several m5-HT_3_AR and GlyR structures [10-12,16,19], identify notable differences between the key residues/motifs for the ECD/TMD cross-talk in the classical CLRs and those in ZAC (Fig. 10A). Yet, these differences do not render the ZAC ECD and TMD-ICD unable to engage in cross-talk with their respective complementary hα_1_ GlyR and m5-HT_3_AR domains. While key residues located in the β6-β7 and β8-β9 loops of 5-HT_3_R and nAChR ECDs also are conserved in ZAC, three residues highly conserved in all classical CLRs (a Glu/Asp in β1-β2 loop, an Arg in pre-M1, and a Tyr/Phe in M1) are not found in ZAC (Fig. 10A) [12]. Of specific interest for ZAC/hα_1_-Gly, the Thr^70^ residue in the β1-β2 loop that interacts with a highly conserved Pro residue in the M2-M3 linker in TMD in α_1_ GlyR, an interaction that has been proposed to be of key importance for its gating [10], is not conserved in ZAC either (Fig. 10A). As evidenced by the lack of a negatively charged residue in β8-β9 of anion-selective CLRs and GLIC and of a negatively charged residue in β1-β2 of ELIC, not all of these residues/motifs need to be present in a CLR to enable ECD/TMD cross-talk (Fig. 10A). Still, it is striking that not less than three of these otherwise highly conserved residues are substituted for residues with distinct physicochemical properties in ZAC, and it seems plausible that these ECD/TMD interface differences could contribute to the distinct gating characteristics exhibited by ZAC compared to classical CLRs [22-24]. Analogously, the “unnatural” ECD/TMD interface interactions in the four chimeric subunits in this study are likely to determine whether functional pentameric complexes can be assembled from them or not. Thus, even though the modifications to the Cys-loop FPX-motif and/or to the C-terminus did not induce functionality into ZAC/m5-HT_3_A and hα_1_-Gly/ZAC (Fig. 4B), it is certainly possible that other modifications to their ECD/TMD interfaces could have yielded functional chimeras. As for the two functional chimeras, the ECD/TMD interface compositions in m5-HT_3_A/ZAC and ZAC/hα_1_-Gly most likely also contribute to their distinct functional properties and channel characteristics compared to their parent CLRs that will be addressed in the following sections.

The 12-fold and 100-fold higher agonist potencies exhibited by 5-HT at m5-HT_3_A/ZAC compared to WT m5-HT_3_AR and by Zn^2+^ at ZAC/hα_1_-Gly compared to WT ZAC, respectively, are truly striking (Fig. 2A and 3A), but the left-shifted agonist concentration-response relationships at the chimeras actually align well with previous findings. While cyste-amine has been reported to display similar or slightly increased EC_50_ values at ELIC/α_7_-nACh chimeras compared to WT ELIC [59], orthosteric agonists have exhibited significantly higher potencies at α_7_-nACh/5-HT_3_A (5–10 fold), 5-HT_3_A/ELIC (3–10 fold) and GLIC/α_1_-Gly (30 fold) chimeras than at their respective ECD-contributing parent receptors [52,57,58], and H^+^ has exhibited a biphasic concentration-response relationship at GLIC/ρ_1_-Gly chimeras with fitted pH_50_ values higher and lower than its pH_50_ at WT GLIC [56]. While the increased agonist potency exhibited by 5-HT at m5-HT_3_A/ZAC could be rooted in the slower desensitization characteristics exhibited by the chimera compared to WT m5-HT_3_AR, the dramatically higher Zn^2+^ potency at ZAC/hα_1_-Gly that at WT ZAC is more difficult to explain. Judging from the agonist profiles at m5-HT_3_A/ZAC and ZAC/hα_1_-Gly, the two chimeras appear to be allosterically stabilised in active conformations compared to WT m5-HT_3_AR and WT ZAC, respectively, a notion corroborated by the significant spontaneous activity exhibited by the chimeras, which at least in the case of ZAC/hα_1_-Gly appears to be of a substantial higher degree than those of its two parent receptors (Fig. 1B, 2B and 3C) [24]. Notably, however, the dramatically increased agonist potency of Zn^2+^ at ZAC/hα_1_-Gly compared to WT ZAC is contrasted by the apparent comparable agonist potencies displayed by H^+^ at two receptors (Fig. 3A). This suggests that the molecular mechanisms underlying Zn^2+^- and H^+^-elicited ZAC gating could differ, but the present data does not warrant elaborate speculations about this.

Just as their pharmacological properties, the channel characteristics exhibited by other ECD/TMD-ICD chimeras derived from distantly related CLRs have often differed considerably from those of their parent receptors [52,56-59]. While the desensitization properties displayed by the two functional chimeras in this study largely resembled those of their respective TMD-ICD-contributing parent receptor (Fig. 2C and 3C), the desensitization characteristics of α_7_-nACh/5-HT_3_A and ELIC/α_7_-nACh chimeras have conversely been reported to be intermediate to those of their parent receptors [52,59]. Moreover, analogously to findings for GLIC/α_1_-Gly and GLIC/ρ_1_-GABA_A_ chimeras [56,57], the robust spontaneous activity exhibited by ZAC/hα_1_-Gly contrasts the lack of constitutive activity in WT hα_1_ GlyR (Fig. 3B). One interpretation of this would be that the molecular determinants for the spontaneous activity exhibited by WT ZAC resides within its ECD, but caution is probably in order when extrapolating from the spontaneous activity in a chimera to speculations about the molecular origin of this characteristic in the WT channel. Although the functionalities of these ECD/TMD-ICD chimeras certainly underline the modular nature of the CLR, the energy barriers between conducting and non-conducting states of any CLR will invariably arise from the pentameric complex in its entirety. Thus, the kinetic basis for and the molecular origin of the spontaneous activity exhibited by WT ZAC and one of the two functional chimerast derived from it could be fundamentally different.

### 4.2. Molecular basis for agonist-induced ZAC activation

Collectively, the robust currents elicited by Zn^2+^ and H^+^ through ZAC/hα_1_-Gly and the lack of significant agonist activity of them at m5-HT_3_A/ZAC strongly suggests that both agonists induce ZAC activation through its ECD. That said, the black-and-white differences in H^+^ functionality at the two chimeras do not rule out that proton-mediated ZAC gating also could involve protonation of residues (proton sensors) in its TMD. In this connection it is interesting to note that while GLIC/α_1_-Gly and GLIC/ρ_1_-GABA_A_ chimeras analogously to ZAC/hα_1_-Gly are activated by H^+^ [56,57], WT GLIC activation has been proposed to arise from protonation of a Glu residue in the ECD that subsequently triggers channel gating via formation of water-mediated hydrogen-bond networks through electrostatic triads of residues located on both sides of the ECD/TMD interface [82,83]. It is possible that protonation of residue(s) in the ZAC ECD is propagated through the receptor complex and induce channel gating by a similar mechanism, even though the similar agonist potencies displayed by H^+^ at WT ZAC and ZAC/hα_1_-Gly certainly points to the ECD as the key domain. The putative existence of additional Zn^2+^ binding site(s) located in the ZAC TMD seems far less likely. In this connection, the Zn^2+^-mediated inhibition of 5-HT-evoked signalling through m5-HT_3_A/ZAC is interesting (Fig. 2B), albeit interpretations about the molecular origin of this inhibition are complicated by the fact that Zn^2+^ targets both parent receptors of this chimera. To our knowledge, the location of the Zn^2+^ binding site(s) in m5-HT_3_AR has not been identified, but given that Zn^2+^ certainly seems to target the ECD of ZAC and that the metal ion exhibits comparable antagonist potencies at the chimera and at WT m5-HT_3_AR, it seem more plausible that the metal ion mediates its inhibition of this chimera through a site in the ECD (m5-HT_3_A) than in the TMD-ICD (ZAC).

The importance of the ECD for Zn^2+^- and H^+^-mediated gating of ZAC could be argued to align well with the location of the orthosteric site in this domain of the classical CLR [1,6,18]. However, considering the vastly different sizes and physicochemical properties of the two ZAC agonists (and of Cu^2+^) compared to ACh, 5-HT, GABA and Gly and the conserved overall structural architecture of the orthosteric site in the classical CLRs, it seems unlikely that the three ZAC agonists act through the corresponding site in ZAC. It is obviously not surprising that the residues in loops A-F forming interactions with ACh, 5-HT, GABA or Gly in the classical CLRs are not conserved in ZAC (Fig. 10B). However, these six loops in ZAC also only comprise four candidate residues for putative Zn^2+^ binding and protonation (for the latter: residues with sidechain pK_a_ of 4–7); Glu^100^ (loop A) and Asp^116^, Asp^118^ and His^120^ (loop E). According to our homology model the former residue is located too far apart from other candidate residues to accommodate Zn^2+^ binding, and the latter three residues have been investigated in the mutagenesis study (Figs. 5 and 10B).

Given the unlikely involvement of loops A-F in Zn^2+^ binding to the ZAC ECD, we attempted to identify its binding site(s) by an elaborate alanine scan of putative candidate residues in this domain (section 3.2). Since none of the introduced mutations in ZAC resulted in significantly reduced agonist potency for Zn^2+^ at the receptor, we can not claim to have identified this site. Assuming that the ZAC ECD indeed does comprise Zn^2+^ binding site(s), there thus has to be alternative explanations for these negligible effects of the introduced mutations. As Zn^2+^ typically will coordinate to the side-chains of 3 or 4 residues in its protein binding [70-74], the lack of significant effect of a mutation of one of four coordinating residues on Zn^2+^ binding affinity could perhaps be ascribed to spatial rearrangement of the remaining Zn^2+^-coordinating residues or to substitution of the mutated residue in the binding site for another candidate Zn^2+^-binding residue located in its proximity, thus compensating for the lost interaction in the mutant. Cluster 1 in particular comprises several candidate Zn^2+^-binding residues positioned with inter-residual distances that in addition to making it feasible for them to form the Zn^2+^ binding themselves also potentially could substitute for a mutated other residue in a proximate site (Table 2). However, it seems unlikely that several of Zn^2+^ site-forming residues could be mutated without any of the mutations causing substantial impairment of its binding affinity, and the fact that Zn^2+^ exhibited WT-like agonist functionality at several mutants comprising double or triple mutations of proximate residues within a cluster also challenges this hypothesis. Another possibility is that the ECD comprises more than one Zn^2+^ binding site, and that metal ion binding to either one of these sites is sufficient to induce ZAC activation. However, this would entail that Zn^2+^ potency at ZAC is unaffected by the elimination/impairment of either of these distinct binding sites, which also seems improbable. Finally, in view of the meagre outcome of the mutagenesis study, we obviously can not completely rule out that some of the ECD candidate residues not included in the mutagenesis study, be it the few residues in the four defined clusters that were not subjected to mutagenesis or the five “non-cluster” residues, could be involved in Zn^2+^ binding to ZAC. All in all, however, none of these explanations are particular satisfactory, and in light of the negligible effects of all tested mutations we are admittedly puzzled about how Zn^2+^ mediates its ZAC activation.

Although the exact location of Zn^2+^ binding site(s) and the residue(s) involved in H^+^-evoked gating of ZAC thus remains to be determined, both agonists are bound to act through site(s)/residue(s) in other ZAC ECD regions than that corresponding to the orthosteric site in the classical CLR. This in turn raises the questions of whether Zn^2+^ and H^+^ can be considered the orthosteric agonists of ZAC, and whether they in fact act as pure agonists at the receptor. It is a distinct possibility that an yet unidentified endogenous ligand targets the ZAC ECD subunit interface cavity formed by loops A-F and thus constitutes the true orthosteric ZAC agonist, which per definition this would make Zn^2+^, Cu^2+^ and H^+^ allosteric ligands. Considering that Zn^2+^, Cu^2+^ and H^+^ act as pure allosteric modulators without significant intrinsic activity at other CLRs [28,29,31-40], it is thus tempting to speculate that the ZAC signalling evoked by Zn^2+^ and H^+^ in fact could arise from them acting as positive allosteric modulators (PAMs) or allosteric agonists/PAMs (ago-PAMs) on the significant inherent constitutive activity of the ZAC complex.

### 4.3. Functional importance of Leu9′ for ZAC function

In concordance with previous findings of the key importance of the M2 Leu9′ residue for classical CLR function [77-81,84], ZAC signalling was substantially altered by introduction of four conservative mutations of this residue (Figs. 8 and 9). The specific impact of a L9′X mutation on functional properties and channel characteristics will inevitably be closely linked to the inherent properties of the channel it is introduced in. Thus, introduction of conservative L9′X mutations in all five subunits of α_7_ and muscle-type nAChRs have yielded dramatically decreased desensitization kinetics and left-shifted agonist concentration-response relationships [77-79], whereas L9′X mutations in other CLRs have mediated more subtle changes in these two properties while changing other kinetic properties and still inducing spontaneous activity in the receptors [81,84]. The aim of the ZAC^L9′x^ mutants was thus to probe the impact of changes to the intermolecular interactions forming the leucine ring in a channel that already exhibits atypical characteristics compared to other CLRs

Although its low functional expression made it somewhat of an outlier, ZAC^L9′A^ overall exhibited very similar characteristics to those of the other three ZAC^L9′X^ mutants, with the WT-like Zn^2+^ agonist potency as its only distinctive property (Figs. 8 and 9). Together with L9′T, the L9′A mutation represent the least conservative of the four Leu9′ substitutions, and while the substitution of the isobutyl group of Leu9′ for a methyl group does not seem to impact ZAC functionality differently than the other three L9′X mutations, it may impair the ability of the ZAC^L9′A^ subunit proteins to be expressed as pentameric assemblies at the cell surface of the oocyte. ZAC^L9′V^, ZAC^L9′I^ and ZAC^L9′T^ all exhibited substantially (8–30 fold) left-shifted Zn^2+^ concentration-relationships, and all four L9′X mutants displayed robust increases in spontaneous activity compared to WT ZAC (Fig. 8). Interestingly, removal of the isobutyl group of Leu9′ (L9′A) as well as the substitution of it for other branched-chain aliphatic (L9′V, L9′I) or polar hydrophilic (L9′T) residues induced very similar degrees of constitutive activity, with all four mutants displaying I_100 μM TC_/(I_Zn2+ max_ + I_100 μM TC_) ratios of 34–40% (Fig. 8D, *left*). This “all-or-nothing” pattern indicates that the structural requirements to the Leu9’ sidechain in terms of forming the intermolecular interactions in the leucine ring stabilizing the resting conformations are very strict, since even slight modifications to the sidechain and impairment of these interactions result in a pronounced displacement of the equilibrium between non-conducting and conducting conformations towards the latter. As evidenced by the I_100 μM TC_/(I_Zn2+ max_ + I_100 μM TC_) ratios and the robust Zn^2+^-induced currents through the four mutants, the majority of the ZAC^L9’X^ receptors in the oocyte still exist in resting conformation(s), and it is possible that other less conservative L9’X mutations could have induced even higher degrees of spontaneous activity in the channel. However, the present data certainly illustrate the dramatically altered energy barriers between non-conducting and conducting states in the ZAC^L9’X^ mutants and the key importance of Leu9’ for the conformational transitions of ZAC, something also evident from the substantially slower activation, deactivation and desensitization kinetics displayed by the ZAC^L9’X^ receptors compared to WT ZAC (Fig. 9).

In conclusion, the functionality of the m5-HT_3_A/ZAC and ZAC/hα_1_-Gly chimeras and the dramatical impact of L9′X mutations on ZAC signalling properties strongly indicate that the molecular mechanisms underlying signal transduction through ZAC resemble those of the classical CLRs. The ECD appears to be the key domain for Zn^2+^- and H^+^-mediated ZAC gating, but the exact locations of the metal ion binding site(s) and the proton sensor(s) within this domain still remain to be delineated. While ZAC thus is an atypical CLR in terms of its (identified) agonists and channel characteristics, its signal transduction seems to undergo similar conformational transitions as those of the classical CLR.

## Figures and Tables

**Table 1:** Averaged kinetic characteristics of the current traces evoked by sustained (4 min) applications of saturating agonist concentrations in oocytes expressing WT m5-HT_3_AR (100 μM 5-HT), WT ZAC (10 mM Zn^2+^), WT hα_1_ GlyR (100 μM Gly) and the chimeric receptors m5-HT_3_A/ZAC (3 μM 5-HT) and ZAC/hα_1_-Gly (30 μM Zn^2+^). Δt_start-to-peak_: time from the start of the agonist application until peak current is reached (in *s*); Δt_peak-to-plateau_ (WT hα_1_ GlyR and ZAC/hα_1_-Gly): time from the peak current to plateau is reached (in *s*); I_residual, 4 min_: residual current after 4 min of agonist application (in % of peak current). Data are given as mean ± S.E.M. values with the number of traces (n) upon which the respective data are based. Representative traces are given in Fig. 2C and Fig. 3C.

Receptor	Δt_start-to-peak_ (*s*)	Δt_peak-to-plateau_ (*s*)	I_residual, 4 min_ (% of I_peak_)	n
WT m5-HT_3_AR	2.1 ± 0.4	–	~0 (none)	7
m5-HT_3_A/ZAC	46 ± 7.2	–	67 ± 6	5
WT ZAC	4.8 ± 0.5	–	21 ± 3	8
ZAC/hα_1_-Gly	10 ± 1.9	56 ± 3.8	63 ± 6	8
WT hα_1_ GlyR	2.7 ± 0.3	52 ± 5.9	51 ± 5	7

**Table 2:** Inter-residual distances between candidate Zn^2+^-binding residues in Clusters 1, 2, 3 and 4 in the ECD of the ZAC homology model. The distances (given in Å) are measured from the carboxylate carbon atom in Glu and Asp, the sulphur atom in the thiol group of Cys, and the imidazole ring in His residues. For the intramolecular clusters (1, 2 and 4), distances between the residues within the same subunit are given, and for the inter-molecular cluster 3 both distances between residues within the same subunit as well as the distances between residues in two neighbouring subunits (in italics, underlined) are given. The distances are measured in one subunit (for cluster 3: in two subunits) of the ZAC complex, but the distances are very similar to and thus highly representative for the same distances in the other subunits of the pentamer.

Cluster 1	Asp^69^	His^79^	His^82^	Asp^116^	Asp^118^	His^120^	His^166^
**Asp^39^**	14.1	20.2	19.3	11.9	11.5	6.1	4.9
**Asp^69^**	–	11.9	17.3	12.2	10.7	11.0	18.9
**His^79^**	–	–	8.7	9.9	11.1	13.3	23.9
**His^82^**	–	–	–	8.2	9.9	12.6	21.4
**Asp^116^**	–	–	–	–	5.9	4.8	14.6
**Asp^118^**	–	–	–	–	–	5.7	15.0
**His^120^**	–	–	–	–	–	–	9.7
	Cluster 3	Glu^24^	Glu^89^	Asp^105^	Asp^108^	Glu^130^	
	**Glu^24^**	*18.2*	6.7	23.7	11.3	28.2	
	**Glu^89^**	*17.2*	*10.5*	17.9	8.3	23.6	
	**Asp^105^**	*24.3*	*17.2*	*5.6*	10.4	8.0	
	**Asp^108^**	*20.4*	*12.8*	*12.0*	*13.0*	14.8	
	**Glu^130^**	*30.4*	*23.8*	*9.4*	*17.8*	*11.4*	
Cluster 2	*His^139^*	*Asp^143^*	*His^144^*		Cluster 4	*Glu^162^*	*Cys^195^*
*Glu^136^*	7.6	6.8	8.5		*Glu^160^*	11.6	15.0
*His^139^*	–	7.4	7.9		*Glu^162^*	–	13.6
*Asp^143^*	–	–	6.8				

## Abbreviations:

5-HT: 5-hydroxytryptamine
5-HT_3_R: 5-HT_3_ receptor
CLR: Cys-loop receptor
ECD: extracellular domain
GABA: *γ*-aminobutyric acid
GABA_A_R: GABA_A_ receptor
GlyR: glycine receptor
nAChR: nicotinic acetylcholine receptor
TEVC: two-electrode voltage clamp
TMD: transmembrane domain
ICD: intracellular domain
ZAC: Zinc-Activated Channel

## References

[1] Taly A, Corringer P-J, Guedin D, Lestage P, Changeux J-P, Nicotinic receptors: allosteric transitions and therapeutic targets in the nervous system, Nat Rev Drug Discov 8 (9) (2009) 733–750. 19721446 10.1038/nrd2927

[2] Bertrand D, Lee C-H, Flood D, Marger F, Donnelly-Roberts D, Esbenshade TA, Therapeutic Potential of α7 Nicotinic Acetylcholine Receptors, Pharmacol Rev 67 (4) (2015) 1025–1073. 26419447 10.1124/pr.113.008581

[3] Grupe M, Grunnet M, Bastlund JF, Jensen AA, Targeting α4β2 nicotinic acetylcholine receptors in central nervous system disorders: perspectives on positive allosteric modulation as a therapeutic approach, Basic Clin Pharmacol Toxicol 116 (3) (2015) 187–200. 25441336 10.1111/bcpt.12361

[4] Walstab J, Rappold G, Niesler B, 5-HT_3_ receptors: role in disease and target of drugs, Pharmacol Ther 128 (1) (2010) 146–169. 20621123 10.1016/j.pharmthera.2010.07.001

[5] Fakhfouri G, Rahimian R, Dyhrfjeld-Johnsen J, Zirak MR, Beaulieu J-M, Witkin JM, 5-HT_3_ receptor antagonists in neurologic and neuropsychiatric disorders: The iceberg still lies beneath the surface, Pharmacol Rev 71 (3) (2019) 383–412. 31243157 10.1124/pr.118.015487

[6] Chua HC, Chebib M, GABA_A_ Receptors and the Diversity in their Structure and Pharmacology, Adv Pharmacol 79 (2017) 1–34. 28528665 10.1016/bs.apha.2017.03.003

[7] Engin E, Benham RS, Rudolph U, An Emerging Circuit Pharmacology of GABA_A_ Receptors, Trends Pharmacol Sci 39 (8) (2018) 710–732. 29903580 10.1016/j.tips.2018.04.003 PMC6056379

[8] Lynch JW, Zhang Y, Talwar S, Estrada-Mondragon A, Glycine Receptor Drug Discovery, Adv Pharmacol 79 (2017) 225–253. 28528670 10.1016/bs.apha.2017.01.003

[9] Cioffi CL, Modulation of glycine-mediated spinal neurotransmission for the treatment of chronic pain, J Med Chem 61 (7) (2018) 2652–2679. 28876062 10.1021/acs.jmedchem.7b00956

[10] Du J, Lü W, Wu S, Cheng Y, Gouaux E, Glycine receptor mechanism elucidated by electron cryo-microscopy, Nature 526 (7572) (2015) 224–229. 26344198 10.1038/nature14853 PMC4659708

[11] Hassaine G, Deluz C, Grasso L, Wyss R, Tol MB, Hovius R, Graff A, Stahlberg H, Tomizaki T, Desmyter A, Moreau C, Li X-D, Poitevin F, Vogel H, Nury H, X-ray structure of the mouse serotonin 5-HT_3_ receptor, Nature 512 (7514) (2014) 276–281. 25119048 10.1038/nature13552

[12] Polovinkin L, Hassaine G, Perot J, Neumann E, Jensen AA, Lefebvre SN, Corringer P-J, Neyton J, Chipot C, Dehez F, Schoehn G, Nury H, Conformational transitions of the serotonin 5-HT_3_ receptor, Nature 563 (7730) (2018) 275–279. 30401839 10.1038/s41586-018-0672-3 PMC6614044

[13] Zhu S, Noviello CM, Teng J, Walsh RM, Kim JJ, Hibbs RE, Structure of a human synaptic GABA_A_ receptor, Nature 559 (7712) (2018) 67–72. 29950725 10.1038/s41586-018-0255-3 PMC6220708

[14] Morales-Perez CL, Noviello CM, Hibbs RE, X-ray structure of the human α4β2 nicotinic receptor, Nature 538 (7625) (2016) 411–415. 27698419 10.1038/nature19785 PMC5161573

[15] Kumar A, Basak S, Rao S, Gicheru Y, Mayer ML, Sansom MSP, Chakrapani S, Mechanisms of activation and desensitization of full-length glycine receptor in lipid nanodiscs, Nat Commun 11 (1) (2020), 10.1038/s41467-020-17364-5. PMC7385131 32719334

[16] Yu J, Zhu H, Lape R, Greiner T, Du J, Lü W, Sivilotti L, Gouaux E, Mechanism of gating and partial agonist action in the glycine receptor, Cell 184 (4) (2021) 957–968.e21. 33567265 10.1016/j.cell.2021.01.026 PMC8115384

[17] Noviello CM, Gharpure A, Mukhtasimova N, Cabuco R, Baxter L, Borek D, Sine SM, Hibbs RE, Structure and gating mechanism of the α7 nicotinic acetylcholine receptor, Cell 184 (8) (2021) 2121–2134.e13. 33735609 10.1016/j.cell.2021.02.049 PMC8135066

[18] Kim JJ, Hibbs RE, Direct Structural Insights into GABA_A_ Receptor Pharmacology, Trends Biochem Sci 46 (6) (2021) 502–517. 33674151 10.1016/j.tibs.2021.01.011 PMC8122054

[19] Basak S, Gicheru Y, Rao S, Sansom MSP, Chakrapani S, Cryo-EM reveals two distinct serotonin-bound conformations of full-length 5-HT_3A_ receptor, Nature 563 (7730) (2018) 270–274. 30401837 10.1038/s41586-018-0660-7 PMC6237196

[20] Gielen M, Corringer P-J, The dual-gate model for pentameric ligand-gated ion channels activation and desensitization, J Physiol 596 (10) (2018) 1873–1902. 29484660 10.1113/JP275100 PMC5978336

[21] Auerbach A, The energy and work of a ligand-gated ion channel, J Mol Biol 425 (9) (2013) 1461–1475. 23357172 10.1016/j.jmb.2013.01.027 PMC4407511

[22] Davies PA, Wang W, Hales TG, Kirkness EF, A novel class of ligand-gated ion channel is activated by Zn^2+^, J Biol Chem 278 (2) (2003) 712–717. 12381728 10.1074/jbc.M208814200

[23] Trattnig SM, Gasiorek A, Deeb TZ, Ortiz EJC, Moss SJ, Jensen AA, Davies PA, Copper and protons directly activate the zinc-activated channel, Biochem Pharmacol 103 (2016) 109–117. 26872532 10.1016/j.bcp.2016.02.004 PMC5119521

[24] Madjroh N, Davies PA, Smalley JL, Kristiansen U, Söderhielm PC, Jensen AA, Delineation of the functional properties exhibited by the Zinc-Activated Channel (ZAC) and its high-frequency Thr128Ala variant (rs2257020) in *Xenopus* oocytes, Pharmacol Res 169 (2021) 105653, 10.1016/j.phrs.2021.105653. 33962015 PMC8248299

[25] Houtani T, Munemoto Y, Kase M, Sakuma S, Tsutsumi T, Sugimoto T, Cloning and expression of ligand-gated ion-channel receptor L2 in central nervous system, Biochem Biophys Res Commun 335 (2) (2005) 277–285. 16083862 10.1016/j.bbrc.2005.07.079

[26] Chang Y, Modulators of Zinc Activated Channel (US 2019/0022121 A1), Dignity Health, Phoeniz, AZ, United States, 2019.

[27] Mathie A, Sutton GL, Clarke CE, Veale EL, Zinc and copper: pharmacological probes and endogenous modulators of neuronal excitability, Pharmacol Ther 111 (3) (2006) 567–583. 16410023 10.1016/j.pharmthera.2005.11.004

[28] Hosie AM, Dunne EL, Harvey RJ, Smart TG, Zinc-mediated inhibition of GABA_A_ receptors: discrete binding sites underlie subtype specificity, Nat Neurosci 6 (4) (2003) 362–369. 12640458 10.1038/nn1030

[29] Bloomenthal AB, Goldwater E, Pritchett DB, NL. Harrison, Biphasic modulation of the strychnine-sensitive glycine receptor by Zn^2+^, Mol Pharmacol 46 (1994) 1156–1159. 7808436

[30] Kim H, Macdonald RL, An N-terminal histidine is the primary determinant of α subunit-dependent Cu^2+^ sensitivity of αβ3γ2L GABA_A_ receptors, Mol Pharmacol 64 (5) (2003) 1145–1152. 14573764 10.1124/mol.64.5.1145

[31] Laube B, Kuhse J, Rundstrom N, Kirsch J, Schmieden V, Betz H, Modulation by zinc ions of native rat and recombinant human inhibitory glycine receptors, J Physiol 83 (1995) 613–619. 10.1113/jphysiol.1995.sp020610 PMC1157806 7776247

[32] Krishek BJ, Moss SJ, Smart TG, Interaction of H^+^ and Zn^2+^ on recombinant and native rat neuronal GABA_A_ receptors, J Physiol 507 (Pt 3) (1998) 639–652. 9508826 10.1111/j.1469-7793.1998.639bs.x PMC2230811

[33] Gill CH, Peters JA, Lambert JJ. An electrophysiological investigation of the properties of a murine recombinant 5-HT3 receptor stably expressed in HEK 293 cells. Br J Pharmacol 1995;114:1211–21. 7620711 10.1111/j.1476-5381.1995.tb13335.x PMC1510359

[34] Harvey RJ, Thomas P, James CH, Wilderspin A, Smart TG, Identification of an inhibitory Zn^2+^ binding site on the human glycine receptor α1 subunit, J Physiol 520 (1) (1999) 53–64. 10517800 10.1111/j.1469-7793.1999.00053.x PMC2269571

[35] Chen Z, Dillon GH, Huang R, Molecular determinants of proton modulation of glycine receptors, J Biol Chem 279 (2) (2004) 876–883. 14563849 10.1074/jbc.M307684200

[36] Lovinger DM, Inhibition of 5-HT_3_ Receptor-Mediated Ion Current by Divalent Metal Cations in NCB-20 Neuroblastoma Cells, J Neurophysiol 66 (4) (1991) 1329–1337. 1722246 10.1152/jn.1991.66.4.1329

[37] Hubbard PC, Lummis SC. Zn2+ enhancement of the recombinant 5-HT3 receptor is modulated by divalent cations. Eur J Pharmacol 2000;394 189–97. 10771284 10.1016/s0014-2999(00)00143-6

[38] Feng H-J, Macdonald RL, Proton modulation of α_1_β_3_δ GABA_A_ receptor channel gating and desensitization, J Neurophysiol 92 (3) (2004) 1577–1585. 15152020 10.1152/jn.00285.2004

[39] Moroni M, Vijayan R, Carbone A, Zwart R, Biggin PC, Bermudez I, Non-agonist-binding subunit interfaces confer distinct functional signatures to the alternate stoichiometries of the α4β2 nicotinic receptor: an α4-α4 interface is required for Zn^2+^ potentiation, J Neurosci 28 (2008) 6884–6894. 18596163 10.1523/JNEUROSCI.1228-08.2008 PMC3844799

[40] Palma E, Maggi L, Miledi R, Eusebi F, Effects of Zn^2+^ on wild type and mutant neuronal α7 nicotinic receptors, Proc Natl Acad Sci USA 95 (1998) 10246–10250. 9707632 10.1073/pnas.95.17.10246 PMC21493

[41] Bocquet N, Prado de Carvalho L, Cartaud J, Neyton J, Le Poupon C, Taly A, Grutter T, Changeux J-P, Corringer P-J, A prokaryotic proton-gated ion channel from the nicotinic acetylcholine receptor family, Nature 445 (7123) (2007) 116–119. 17167423 10.1038/nature05371

[42] Beg AA, Ernstrom GG, Nix P, Davis MW, Jorgensen EM, Protons act as a transmitter for muscle contraction in C. elegans, Cell 132 (2008) 149–160. 18191228 10.1016/j.cell.2007.10.058 PMC2258244

[43] Feingold D, Starc T, O’Donnell MJ, Nilson L, Dent JA, The orphan pentameric ligand-gated ion channel pHCl-2 is gatedby pH and regulates fluid secretion in *Drosophila* Malpighian tubules, J Exp Biol 219 (2016) 2629–2638. 27358471 10.1242/jeb.141069

[44] Horton RM, Hunt HD, Ho SN, Pullen JK, Pease LR, Engineering hybrid genes without the use of restriction enzymes: gene splicing by overlap extension, Gene 77 (1) (1989) 61–68. 2744488 10.1016/0378-1119(89)90359-4

[45] Pettersen EF, Goddard TD, Huang CC, Couch GS, Greenblatt DM, Meng EC, Ferrin TE, UCSF Chimera–a visualization system for exploratory research and analysis, J Comput Chem 25 (13) (2004) 1605–1612. 15264254 10.1002/jcc.20084

[46] Bohler S, Gay S, Bertrand S, Corringer PJ, Edelstein SJ, Changeux J-P, Bertrand D, Desensitization of neuronal nicotinic acetylcholine receptors conferred by N-terminal segments of the β2 subunit, Biochemistry 40 (7) (2001) 2066–2074. 11329274 10.1021/bi0020022

[47] Olsen JA, Kastrup JS, Peters D, Gajhede M, Balle T, Ahring PK, Two distinct allosteric binding sites at α4β2 nicotinic acetylcholine receptors revealed by NS206 and NS9283 give unique insights to binding activity-associated linkage at Cys-loop receptors, J Biol Chem 288 (50) (2013) 35997–36006. 24169695 10.1074/jbc.M113.498618 PMC3861648

[48] Hoestgaard-Jensen K, Dalby NO, Krall J, Hammer H, Krogsgaard-Larsen P, Frølund B, Jensen AA, Probing α4βδ GABA_A_ receptor heterogeneity: differential regional effects of a functionally selective α4β1δ/α4β3δ receptor agonist on tonic and phasic inhibition in rat brain, J Neurosci 34 (49) (2014) 16256–16272. 25471566 10.1523/JNEUROSCI.1495-14.2014 PMC6608486

[49] Gasiorek A, Trattnig SM, Ahring PK, Kristiansen U, Frølund B, Frederiksen K, Jensen AA, Delineation of the functional properties and the mechanism of action of TMPPAA, an allosteric agonist and positive allosteric modulator of 5-HT_3_ receptors, Biochem Pharmacol 110–111 (2016) 92–108. 10.1016/j.bcp.2016.04.004 27086281

[50] Lansdell SJ, Sathyaprakash C, Doward A, Millar NS, Activation of human 5-hydroxytryptamine type 3 receptors via an allosteric transmembrane site, Mol Pharmacol 87 (1) (2015) 87–95. 25338672 10.1124/mol.114.094540

[51] Steinbach JH, Bracamontes J, Yu Li, Zhang P, Covey DF, Subunit-specific action of an anticonvulsant thiobutyrolactone on recombinant glycine receptors involves a residue in the M2 membrane-spanning region, Mol Pharmacol 58 (1) (2000) 11–17. 10860922 10.1124/mol.58.1.11

[52] Eiselé J-L, Bertrand S, Galzi J-L, Devillers-Thiéry A, Changeux J-P, Bertrand D, Chimaeric nicotinic-serotonergic receptor combines distinct ligand binding and channel specificities, Nature 366 (6454) (1993) 479–483. 8247158 10.1038/366479a0

[53] Dineley KT, Patrick JW, Amino acid determinants of α7 nicotinic acetylcholine receptor surface expression, J Biol Chem 275 (18) (2000) 13974–13985. 10788524 10.1074/jbc.275.18.13974

[54] Mihic SJ, Ye Q, Wick MJ, Koltchine VV, Krasowski MD, Finn SE, Mascia MP, Valenzuela CF, Hanson KK, Greenblatt EP, Harris RA, Harrison NL, Sites of alcohol and volatile anaesthetic action on GABA_A_ and glycine receptors, Nature 389 (6649) (1997) 385–389. 9311780 10.1038/38738

[55] Baker ER, Zwart R, Sher E, Millar NS, Pharmacological properties of the α9α10 nicotinic acetylcholine receptors revealed by heterologous expression of subunit chimeras, Mol Pharmacol 65 (2004) 453–460. 14742688 10.1124/mol.65.2.453

[56] Ghosh B, Tsao T-W, Czajkowski C, A chimeric prokaryotic-eukaryotic pentameric ligand gated ion channel reveals interactions between the extracellular and transmembrane domains shape neurosteroid modulation, Neuropharmacology 125 (2017) 343–352. 28803966 10.1016/j.neuropharm.2017.08.007 PMC5600277

[57] Duret G, Van Renterghem C, Weng Y, Prevost M, Moraga-Cid G, Huon C, Sonner JM, Corringer P-J, Functional prokaryotic-eukaryotic chimera from the pentameric ligand-gated ion channel family, Proc Natl Acad Sci U S A 108 (29) (2011) 12143–12148. 21730130 10.1073/pnas.1104494108 PMC3141993

[58] Price KL, Lummis SCR, Characterization of a 5-HT_3_-ELIC Chimera Revealing the Sites of Action of Modulators, ACS Chem Neurosci 9 (6) (2018) 1409–1415. 29508995 10.1021/acschemneuro.8b00028

[59] Tillman TS, Seyoum E, Mowrey DD, Xu Y, Tang P, ELIC-α7 nicotinic acetylcholine receptor (α7nAChR) chimeras reveal a prominent role of the extracellular-transmembrane domain interface in allosteric modulation, J Biol Chem 289 (20) (2014) 13851–13857. 24695730 10.1074/jbc.M113.524611 PMC4022858

[60] Paulsen IM, Martin IL, Dunn SM. Isomerization of the proline in the M2-M3 linker is not required for activation of the human 5-HT3A receptor. J Neurochem 2009; 110:870–8. 19457066 10.1111/j.1471-4159.2009.06180.x

[61] Stevens RJN, Rüsch D, Davies PA, Raines DE, Molecular properties important for inhaled anesthetic action on human 5-HT3A receptors, Anesth Analg 100 (6) (2005) 1696–1703. 15920198 10.1213/01.ANE.0000151720.36988.09 PMC4533112

[62] Price KL, Bower KS, Thompson AJ, Lester HA, Dougherty DA, Lummis SCR, A hydrogen bond in loop A is critical for the binding and function of the 5-HT3 receptor, Biochemistry 47 (24) (2008) 6370–6377. 18498149 10.1021/bi800222n PMC2649372

[63] Ladefoged LK, Munro L, Pedersen AJ, Lummis SCR, Bang-Andersen B, Balle T, Schiøtt B, Kristensen AS, Modeling and mutational analysis of the binding mode for the multimodal antidepressant drug vortioxetine to the human 5-HT_3A_ receptor, Mol Pharmacol 94 (6) (2018) 1421–1434. 30257860 10.1124/mol.118.113530

[64] i Uki M, Narahashi T, Modulation of serotonin-induced currents by metals in mouse neuroblastoma cells, Arch Toxicol 70 (10) (1996) 652–660. 8870959 10.1007/s002040050325

[65] Pistis M, Belelli D, Peters JA, Lambert JJ, The interaction of general anaesthetics with recombinant GABA_A_ and glycine receptors expressed in *Xenopus* laevis oocytes: a comparative study, Br J Pharmacol 122 (1997) 1707–1719. 9422818 10.1038/sj.bjp.0701563 PMC1565119

[66] Jensen AA, Bergmann ML, Sander T, Balle T, Ginkgolide X is a potent antagonist of anionic Cys-loop receptors with a unique selectivity profile at glycine receptors, J Biol Chem 285 (13) (2010) 10141–10153. 20106969 10.1074/jbc.M109.079319 PMC2843176

[67] De Saint JD, David-Watine B, Korn H, Bregestovski P, Activation of human α1 and α2 homomeric glycine receptors by taurine and GABA, J Physiol 535 (3) (2001) 741–755. 11559772 10.1111/j.1469-7793.2001.t01-1-00741.x PMC2278820

[68] Gielen M, Thomas P, Smart TG, The desensitization gate of inhibitory Cys-loop receptors, Nat Commun 6 (2015) 6829. 25891813 10.1038/ncomms7829 PMC4410641

[69] Bouzat C, Bartos M, Corradi J, Sine SM, The Interface between extracellular and transmembrane domains of homomeric Cys-Loop receptors governs open-channel lifetime and rate of desensitization, J Neurosci 28 (31) (2008) 7808–7819. 18667613 10.1523/JNEUROSCI.0448-08.2008 PMC3844810

[70] Alberts IL, Nadassy K, Wodak SJ, Analysis of zinc binding sites in protein crystal structures, Protein Sci 7 (8) (1998) 1700–1716. 10082367 10.1002/pro.5560070805 PMC2144076

[71] Christianson DW, Structural Biology of Zinc, Adv Protein Chem 42 (1991) 281–355. 1793007 10.1016/s0065-3233(08)60538-0

[72] Daniel AG, Farrell NP, The dynamics of zinc sites in proteins: electronic basis for coordination sphere expansion at structural sites, Metallomics 6 (12) (2014) 2230–2241. 25329367 10.1039/c4mt00213j

[73] Simonson T, Calimet N, Cys_x_His_y_-Zn^2+^ interactions: Thiol vs, Thiolate Coordination. Proteins 49 (1) (2002) 37–48. 12211014 10.1002/prot.10200

[74] Holm RH, Kennepohl P, Solomon EI. Structural and Functional Aspects of Metal Sites in Biology. Chem Rev 1996;96 2239–314. 11848828 10.1021/cr9500390

[75] Madjroh N, Mellou E, Davies PA, Söderhielm PC, Jensen AA, Discovery and functional characterization of N-(thiazol-2-yl)-benzamide analogs as the first class of selective antagonists of the Zinc-Activated Channel (ZAC), Biochem Pharmacol. 193 (2021), 114782, 10.1016/j.bcp.2021.114782. 34560054 PMC9979163

[76] Schofield CM, Jenkins A, Harrison NL, A highly conserved aspartic acid residue in the signature disulfide loop of the α1 subunit is a determinant of gating in the glycine receptor, J Biol Chem 278 (36) (2003) 34079–34083. 12826676 10.1074/jbc.M302416200

[77] Revah F, Bertrand D, Galzi J-L, Devillers-Thiéry A, Mulle C, Hussy N, Bertrand S, Ballivet M, Changeux J-P, Mutations in the channel domain alter desensitization of a neuronal nicotinic receptor, Nature 353 (6347) (1991) 846–849. 1719423 10.1038/353846a0

[78] Labarca C, Nowak MW, Zhang H, Tang L, Deshpande P, Lester HA, Channel gating governed symmetrically by conserved leucine residues in the M2 domain of nicotinic receptors, Nature 376 (6540) (1995) 514–516. 7637783 10.1038/376514a0

[79] Filatov GN, White MM, The role of conserved leucines in the M2 domain of the acetylcholine receptor in channel gating, Mol Pharmacol 48 (1995) 379–384. 7565616

[80] Bianchi MT, Macdonald RL, Mutation of the 9’ leucine in the GABA_A_ receptor γ_2L_ subunit produces an apparent decrease in desensitization by stabilizing open states without altering desensitized states, Neuropharmacology 41 (6) (2001) 737–744. 11640928 10.1016/s0028-3908(01)00132-0

[81] Yakel JL, Lagrutta A, Adelman JP, North RA, Single amino acid substitution affects desensitization kinetics of the 5-hydroxytryptamine type 3 receptor expressed in *Xenopus* oocytes, Proc Natl Acad Sci USA 90 (1993) 5030–5033. 8506347 10.1073/pnas.90.11.5030 PMC46647

[82] Nemecz Á, Hu H, Fourati Z, Van Renterghem C, Delarue M, Corringer P-J, Dutzler R, Full mutational mapping of titratable residues helps to identify proton-sensors involved in the control of channel gating in the *Gloeobacter violaceus* pentameric ligand-gated ion channel, PLoS Biol 15 (12) (2017) e2004470, 10.1371/journal.pbio.2004470. 29281623 PMC5760087

[83] Hu H, Ataka K, Menny A, Fourati Z, Sauguet L, Corringer P-J, Koehl P, Heberle J, Delarue M, Electrostatics, proton sensor, and networks governing the gating transition in GLIC, a proton-gated pentameric ion channel, Proc Natl Acad Sci U S A 115 (52) (2018) E12172–E12181. 30541892 10.1073/pnas.1813378116 PMC6310827

[84] Chang Y, Weiss DS, Substitutions of the highly conserved M2 leucine create spontaneously opening ρ1 γ-aminobutyric acid receptors, Mol Pharmacol 53 (3) (1998) 511–523. 9495819 10.1124/mol.53.3.511

